# Carrier Mobility, Electrical Conductivity, and Photovoltaic Properties of Ordered Nanostructures Assembled from Semiconducting Polymers

**DOI:** 10.3390/ma18194580

**Published:** 2025-10-02

**Authors:** Maria Pop, Ioan Botiz

**Affiliations:** 1 Interdisciplinary Research Institute on Bio-Nano-Sciences, Babeș-Bolyai University, 400271 Cluj-Napoca, Romania; 2 Department of Physics of Condensed Matter and Advanced Technologies, Faculty of Physics, Babeș-Bolyai University, 400084 Cluj-Napoca, Romania

**Keywords:** semiconducting polymers, material processing, ordered nanostructures, carrier mobility, electrical conductivity, photovoltaic properties

## Abstract

Nanostructures composed of semiconducting polymers that adopt highly ordered molecular arrangements at the nano- and microscale typically exhibit enhanced optoelectronic properties. In this study, we aim to establish a comprehensive correlation between nanostructures with varying degrees of molecular order—fabricated using diverse processing methods—and their tailored optoelectronic properties, as demonstrated by various energy devices. These properties include carrier mobility, electrical conductivity, and photovoltaic capabilities measured predominantly in films tens to hundreds of nanometers thick based on semiconducting polymers.

## 1. Introduction

Ordered nanostructures of semiconducting polymers are hierarchical macromolecular assemblies exhibiting a high degree of morphological organization at the nanoscale. Typically, polymeric nanostructures are produced through the efficient processing of long molecular chains in solutions, films, or bulk materials. A wide variety of polymeric structures, such as surface relief nanostructures, can be fabricated using various top-down nanolithography techniques [1,2,3,4,5] and subsequently applied in numerous technological applications [6,7,8,9,10]. However, these nanostructures do not always exhibit a high degree of internal molecular order. An alternative approach is the bottom-up strategy, which relies on physical processes such as polymer microphase separation [11,12,13,14], polymer self-assembly [15,16,17,18,19,20], or polymer crystallization [21,22,23,24,25,26]. This method can generate polymeric nanostructures with high molecular order at the nanoscale directly from molecular building-blocks [4,15,16,27,28]. Consequently, the resulting nanostructures typically exhibit increased crystallinity, reduced defect densities, and fewer grain boundaries. They can take various morphologies, including nanowires [29,30,31,32], nanofibers [33,34,35,36,37], nanotubes [38,39], single crystals [28,30,40,41], lamellar nanostructures [42,43,44,45,46], cylinders [47,48,49,50,51], nanoparticles [52,53,54,55,56], gyroids [47], vesicles [57,58,59,60], micelles [46,61,62,63,64], helix-resembling [53,65,66] or spherical [44] nanoassemblies, among others [67,68]. These ordered structures are primarily formed in thin polymeric films with thicknesses ranging from tens to a few hundreds of nanometers, and they cover varying surface areas, depending on the fabrication method and physical parameters used to produce the semiconducting polymer films.

Achieving a high degree of internal order, particularly in semiconducting nanostructures, is critical because it determines—according to the well-established structure-property relationships in materials science [69,70,71,72,73,74,75,76]—the final optoelectronic properties of the entire ensemble of macromolecules forming a specific nanostructure. These properties may include enhanced or tuned light absorption [77,78,79], and emission [36,80,81,82], optimized charge transfer [83,84,85], improved charge mobility and electrical transport properties [86,87,88,89,90], and favorable electronic interactions [86,91]. In turn, these characteristics have a crucial impact on the overall efficiency of OSCs [92,93,94], OFETs [29,95,96], OLEDs [81], sensors [97,98], and other applications [2,98].

In this review, we aim to connect recent advancements in charge carrier mobility, electrical conductivity, and power conversion efficiency to the types of assembled (ordered) nanostructures composed of semiconducting polymers used in various energy devices—that is, to the molecular arrangements at the nanoscale. Our focus will primarily be on polymer-based semiconducting nanostructures obtained, though not exclusively, through bottom-up methodologies, particularly via self-assembly, phase separation, and crystallization processes.

## 2. The Structure-Processing-Property Relationship and Charge Transport Mechanisms in Semiconducting Polymers

**Figure 1:**
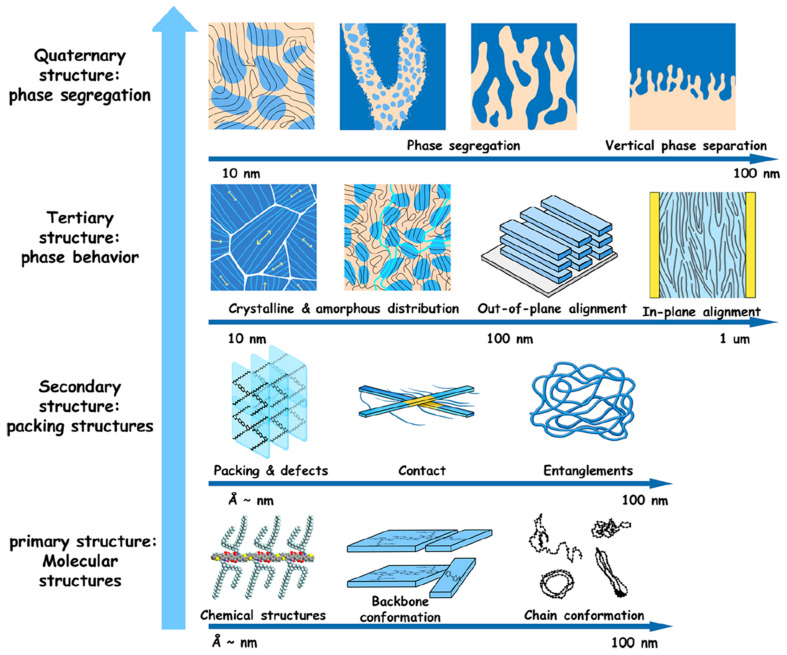
Various multiscale microstructures of semiconducting polymers that can be generated at different size scales. Reproduced from ref. [99] [Copyright (2023), with permission from the American Chemical Society].

Nowadays, it is widely accepted that the primary governing principle in the field of organic optoelectronics is the structure–processing–property relationship [69,70,71,72,73,74,75,76]. This concept states that the final optoelectronic properties of semiconducting materials directly result from the molecularly ordered arrangements adopted by the semiconducting macromolecules at the nano- and microscales during processing. Such arrangements are possible due to polymers’ ability to exhibit various degrees of conformational freedom, typically induced by π-π interactions, side-chain interactions, or even chain entanglement and folding. The hierarchical multiscale structures encompass levels ranging from the nanoscale chemical structure, backbone, and chain conformation to molecular (lamellar, π–π) packing/aggregation and chain entanglements; to amorphous (lacking molecular packing) and crystalline (characterized by high and periodic molecular packing) film microdomains of various orientations and grain boundaries; and finally, to phase-segregated or phase-separated structures in various multicomponent polymer-based blends (Figure 1) [99]. These multiscale structures can be generated through various material processing techniques [100] and dictate how charges separate and how charge carriers migrate within a specific organic material. Consequently, they have a significant impact on charge transport efficiency. Therefore, (i) precise optimization of the polymeric chain orientation with respect to the substrate, (ii) stacking efficiency within various aggregates and crystallites, (iii) intra- and interconnections between aggregates and crystallites, (iv) type and abundance of grain boundaries and tie chains promote the delocalization of charges [86]. This, in turn, can lead to a significant enhancement of charge carrier transport, thereby favoring the fabrication of more efficient energy devices.

**Figure 2:**
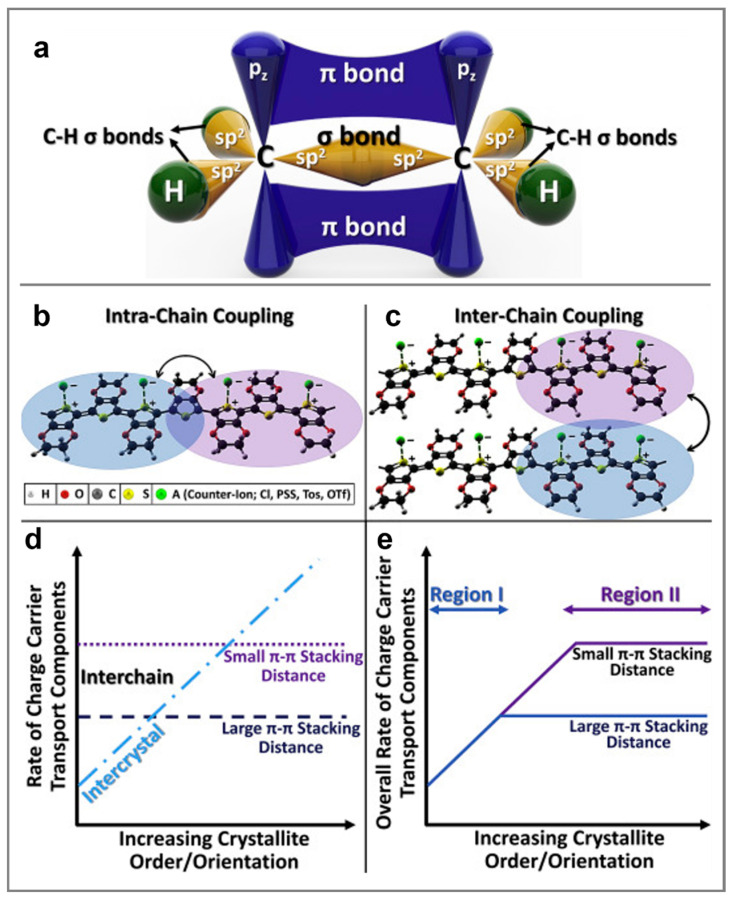
(**a**) An example of schematics depicting the σ and π bonds in a semiconducting polymer. Here, each carbon atom displays three σ bonds (two formed with hydrogen and one with the neighboring carbon atom), and one 2p_z_ orbital that forms π bonds, with the latter being orthogonal to the σ plane and eventually delocalized along the polymer chain. (**b**,**c**) Schematics showing intra- (**a**) and interchain (**b**) coupling in semiconducting polymers. The wave functions of localized charge defects can overlap along a polymer chain, enabling the intrachain coupling. When the distance between individual polymer chains is small, the interchain coupling becomes significant. This interaction enhances the charge transfer integral and increases carrier mobility in conjugated polymers. (**d**) Schematics emphasizing the rates of intercrystallite and interchain charge transport in (semi)conducting polymers. The blue dashed-dot line represents the rate of intercrystallite charge transport as a function of increasing crystallite order or orientation. Charge transport is more efficient at smaller π–π stacking distances (shown by the dotted purple line) compared to larger π–π stacking distances (depicted by the dashed dark-blue line). (**e**) Illustration of the overall rate of charge carrier transport combining both intercrystallite and interchain processes. Here, region I represents the low structural order, while region II stands for the high structural order. Increasing the degree of order in region I enhances the electrical conductivity, while the latter is relatively unaffected by the π–π stacking distance. In contrast, in region II the overall charge transport rate plateaus for both small and large π–π stacking distances, indicating that further improvements in order or orientation have a rather limited impact. However, reducing the π–π stacking distance in region II can still significantly improve the overall charge transport rate and electrical conductivity. Reproduced from ref. [86] (**a**–**e**) [Copyright (2020) the authors, with permission from Elsevier Ltd.].

For example, semiconducting polymers owe their electrical conductivity to their unique backbone structure, characterized by alternating single and double bonds between carbon atoms. Both types of bonds feature localized σ components, while the double bonds also include less localized π bonds (Figure 2a) [86]. When monomers link to form long polymer chains, the repeating pattern of alternating σ and π bonds facilitates the delocalization of π orbitals. This delocalization allows π-electrons to move along the conjugated backbone, promoting various electronic interactions that lead to the formation of bonding and antibonding orbitals throughout the polymer chain [86,91].

It is clear that understanding charge transport in semiconducting polymers is essential for optimizing their material properties and enhancing their performance in various energy devices. A comprehensive understanding of charge transport requires an in-depth knowledge of the transport mechanisms occurring within a single polymer chain, between two or more chains, and at the level of different assemblies of semiconducting chains, such as more or less ordered aggregates and crystallites. Therefore, it is necessary first to understand the interchain and intrachain coupling in semiconducting polymer chains. Intrachain coupling enables charge carriers to move along the polymer backbone through adjacent localized segments. In contrast, interchain coupling facilitates charge hopping between localized sites on different polymer chains or between segments of the same chain that undergo folding (Figure 2b,c) [86]. It is important to emphasize that intrachain coupling is generally stronger than interchain coupling, often resulting in higher charge carrier mobility along the polymer backbone. However, strong interchain coupling plays a crucial role in minimizing charge localization, especially in the presence of structural defects and amorphous regions [86].

Secondly, our goal is to emphasize how aggregates and crystallites, exhibiting a specific degree of molecular order or crystallinity, may impact charge carrier transport. For instance, factors known to enhance intercrystallite charge transport include the degree of order or crystallinity, the size of aggregates or crystallites, their molecular orientation, and the presence of tie chains interconnecting various crystallites [86]. These factors can be controlled by employing the most appropriate processing methodologies, with the primary objective of reducing the π–π stacking distance to generate strong interchain coupling (charge carrier mobility increases as the π–π stacking distance decreases; see Figure 2d) [86]. Conversely, a larger π–π stacking distance typically results in weaker interchain coupling and, consequently, decreased charge carrier mobility. It is important to note that to enhance charge transport, both interchain and intercrystallite charge transport must be optimized through minimization/maximization of the structural disorder/order at the nano- and microscale. While, experimentally, it is possible to reach a limit in crystallite order and orientation (see region II in Figure 2e), additional processing treatments such as doping or the use of additives can often further reduce the π–π stacking distance, thereby improving charge transport even more [86].

**Figure 3:**
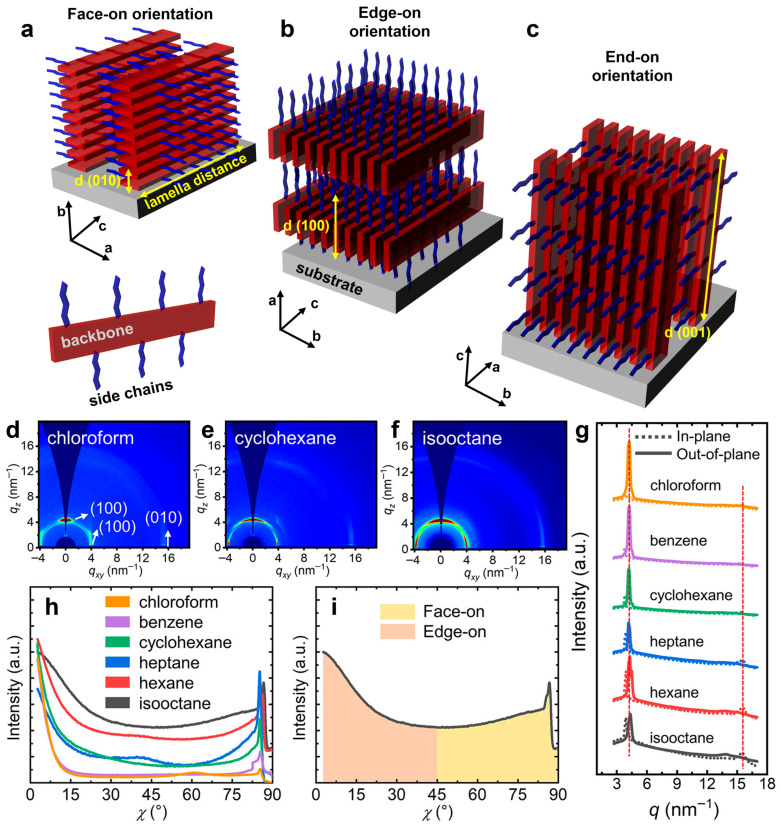
(**a**–**c**) Illustration of different crystallization orientations frequently found in semiconducting polymer thin films: face-on (**a**), edge-on orientation (**b**), and end-on (**c**) orientation. As one can observe, the conjugated backbone is parallel to the surface of substrate in the case of both face-on and edge-on orientations, whereas it is orthogonal with respect to the substrate in the case of end-on orientation. (**d**–**f**) 2D GIWAXS patterns of PCDTPT-ODD films drop-cast from solutions of chloroform (**d**), cyclohexane (**e**), and isooctane (**f**). Here, GIWAXS patterns reveal that in PCDTPT-ODD films drop-cast from chloroform solution the content of edge-on orientations is over 74%, whereas this content decreases below 54% in film analogs drop-cast from isooctane solution (similarly, the content of face-on orientations increases from over 25% to more than 46%). (**g**) 1D GIWAXS plots along the out-of-plane and in-plane directions measured for PCDTPT-ODD films drop-cast from various solutions. (**h**) Intensity distribution of the (100) peak as relative to the azimuth angle χ for various PCDTPT-ODD films. (**i**) Fraction of face-on and edge-on orientations extracted for various PCDTPT-ODD films relative to the χ angle. Reproduced from ref. [87] (**d**–**i**) [Copyright (2021), with permission from the American Chemical Society].

We conclude this section with a brief discussion of the most common orientations observed in semicrystalline assemblies (crystallites) typically formed by semiconducting polymers. These orientations include disordered, face-on, edge-on, and end-on orientations (Figure 3a–c) [86,87]. In both face-on and edge-on orientations, the polymeric backbones are aligned parallel to the substrate surface, whereas in the end-on orientation, the backbones are oriented perpendicular to the substrate. This latter orientation is characteristic of highly ordered structures such as single crystals [28]. These orientations are achieved through various processing techniques, including multiple film casting methods and post-fabrication treatments such as thermal and melt-annealing, solvent vapor annealing (SVA), mechanical shearing, rubbing, stretching, hot pressing, and others [100]. Molecular orientation is typically determined using X-ray scattering and diffraction measurements, as the periodic planes of most atomic or molecular species diffract incident X-ray beams of specific wavelengths (noting that the crystalline orientation is indicated by the direction of the diffracted beam) (Figure 3d–h). Key parameters related to molecular interactions and structure can be extracted from X-ray analysis, including the fraction of edge-on and face-on orientations (Figure 3i), the π–π stacking distance (the distance between the centers of aromatic parallel or offset rings) and the crystallographic d-spacing (distance between parallel planes of atoms or molecules within a crystal lattice). Further details on X-ray and other techniques used to determine preferential orientations in semiconducting polymer films can be found in comprehensive reviews available in the literature [86,99].

Finally, it is important to evaluate the efficiency of charge carrier transport in the aforementioned crystalline orientations. It is now well established that charge transport is anisotropic, with charge carrier mobility generally higher along the conjugated backbone (i.e., intrachain) than along the orthogonal direction of the interchain π–π stacking [90,101,102]. Moreover, the actual landscape within thin films of semiconducting materials is often more complex, characterized by a variety of multiscale structures. Therefore, additional factors and structures must be optimized to enhance overall charge transport and charge carrier mobility in these materials. Of particular importance are grain boundaries—interfaces within a polycrystalline film where two or more crystals with different orientations meet—and the abundance of so-called tie chains that interconnect crystalline or ordered domains, creating continuous pathways for charge carrier transport. Fewer grain boundaries and a higher abundance of tie chains both promote increased charge carrier mobilities [28,87]. More detailed information on the effects of molecular conformations and morphology on charge transport processes in semiconducting polymer films can be found in the literature [87,88,89], along with additional theoretical studies that can relate the processing and multiscale structural order of various semiconducting compounds to their optoelectronic properties and device performance [103].

## 3. Polymeric Semiconducting Nanostructures Displaying High Charge Carrier Mobilities

### 3.1. Carrier Mobility in Diketopyrrolopyrrole-Based Nanostructures

The enhancement of optoelectronic properties in semiconducting polymers—including charge carrier mobility, electrical conductivity, and photovoltaic performance—is crucial for the development of state-of-the-art nanotechnological applications such as OSCs, OFETs, and OLEDs. We begin by emphasizing the ability of semiconducting polymer chains to transport various charges through hopping between chains or traveling along them. More precisely, we relate charge carrier mobility (µ) to the speed at which charge carriers, such as electrons or holes, can move along or between polymer chains relative to a reference direction in the presence of an electric field [104]. As we will observe, this reference direction is typically preferred either along the π–π stacking axis or along the long chain axis, known as the backbone. Moreover, semiconducting polymeric systems generally exhibit high mobility due to cofacial π-electron system to π-electron system interactions, which are also responsible for photoluminescence quenching [105]. More theoretical details on charge carrier mobility in semiconducting polymers can be found in the literature [106]. Finally, it is worth noting that we will primarily focus on recent scientific reports published roughly within the last five years that discuss charge carrier mobility in semiconducting polymers. Earlier studies (prior to 2020) will be briefly mentioned, as the most significant charge mobilities recorded for various polymers have been comprehensively summarized in several extensive reviews available in the literature [86,107,108,109,110]).

Over the past five years, research on charge mobility in semiconducting polymers has predominantly focused on systems based on DPP. This trend has persisted since the 2013–2015 period, when high charge mobilities of µ = 7 cm^2^V^−1^s^−1^ and µ_h_ = 24 cm^2^V^−1^s^−1^ were reported for a PDTTDPP copolymer [111], and a highly crystalline DPPBTSPE system [29], respectively. Both polymers were drop-cast from solutions processed in advance using simple thermal protocols, resulting in highly ordered nanowires. Additionally, a longer DPPBTSPE that produced nanowires with weaker π–π stacking upon processing exhibited a mobility of only 4.15 cm^2^V^−1^s^−1^ [29], underscoring the critical role of molecular packing strength in the π-π direction. Moreover, 24 cm^2^V^−1^s^−1^ remains a record for DPP-based polymers, as subsequent reports of mobilities for over 60 BPP-based polymers did not exceed µ_h_ = 17.8 cm^2^V^−1^s^−1^ [108,109,112,113,114,115]. This latter value was achieved for a DPP-selenophene vinylene selenophene (DPP-SVS) polymer containing linear spacer groups, which was solution-sheared and thermally annealed to form well-interconnected granular nanodomains [115].

**Figure 4:**
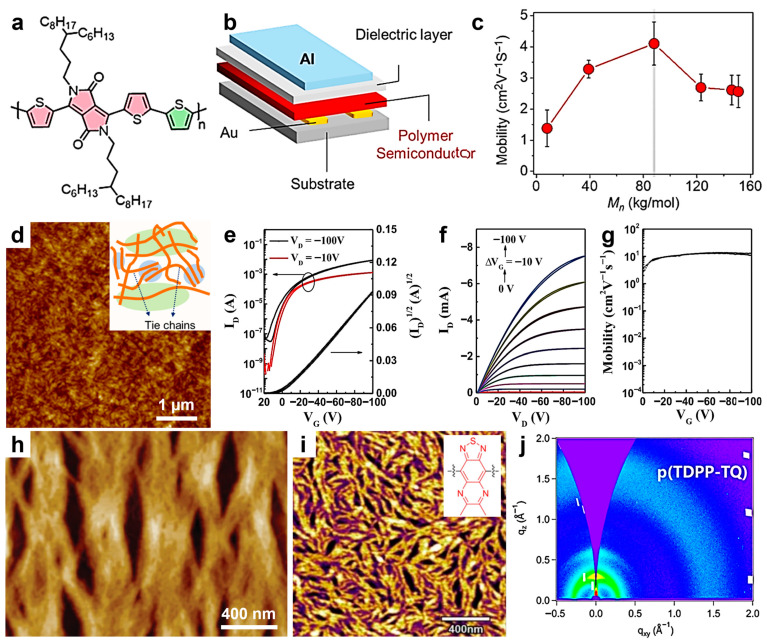
(**a**) Chemical structure of PDPPT3. (**b**) Schematics depicting a typical OFET device in a top-gate/bottom-contact configuration. (**c**) Plot depicting the impact of polymer M_n_ on the hole mobility. (**d**) AFM height image depicting the microstructure of a thin film of PDPPT3. The inset is a schematic representation of the microstructure of PDPPT3 chains of M_n_ = 88 kg/mol, with the emphasis on tie-chains. (**e**,**f**) Typical electrical measurements evaluating the OFET devices made of TDPP-Se (M_n_ = 40.7 kg/mol) and recorded in the parallel direction: transfer (**e**) and output (**f**) characteristics referring to *I_D_* versus *V_G_* at a fixed *V_D_*, and to *I_D_* versus *V_D_* at various fixed *V_G_*, respectively. (**g**) Saturation mobility against *V_G_* in the parallel direction for devices measured in (**e**,**g**). (**h**) AFM topography micrograph depicting a TDPP-Se film bar-coated from D-ODCB solution onto a substrate kept at 120 °C. (**i**) AFM height micrograph showing the fiber-like microstructure of a P(TDPP-TQ) film. (**j**) 2DGIWAXS pattern of P(TDPP-TQ). Reproduced from ref. [114] (**a**–**d**) [Copyright (2020), with permission from the American Chemical Society], ref. [119] (**e**–**h**) [Copyright (2022) WILEY-VCH Verlag GmbH with permission from John Wiley and Sons], and ref. [118] (**i**,**j**) [Copyright (2022) the author(s), with permission from Springer Nature].

Relatively high hole mobilities were also observed (see Table 1) in DPP-2T [113], PDPPy-Se [112], or PDPPT3 (see its chemical structure in Figure 4a) [114]. In these cases, polymer chains tended to pack into strong aggregates, large-scale crystallites, or highly continuous structures interconnected by tie chains, respectively. Notably, the presence of tie chains was reported to yield the highest hole mobility measured in a typical OFET device (Figure 4b) for polymer chains with an intermediate molecular weight of M_n_ = 88 kg/mol (Figure 4c), suggesting the existence of improved transport pathways via tie chains (Figure 4d) [114].

More recently, other DPP-based polymers have been designed and synthesized to fabricate more efficient OFETs. Reports on such polymers have revealed hole mobilities ranging from 2.2 × 10^−3^ to 13.77 cm^2^V^−1^s^−1^ [116,117,118,119,120,121] (see the typical OFET characteristics presented in Figure 4e–g), for films composed of smooth surfaces with nanopinholes [120] or one-dimensional (1D) rod-like nanostructures of tuned molecular weight (Figure 4h) [119]. In contrast, electron mobilities up to 7.76 cm^2^V^−1^s^−1^ were reported for fiber-like domains exhibiting face-on packing and a relatively short π–π stacking distance of 0.370 nm (Figure 4i,j) [118]. Nonetheless, the aforementioned polymers are not particularly suitable for flexible OFETs. Therefore, DPP-based polymers with additional flexible blocks, such as PDPP-TT-PDMS, have been recently designed and optimized [122]. These systems can be processed through solution shearing combined with thermal annealing (TA) to produce highly interconnected fiber-like nanostructures capable of transporting charges with a respectable mobility even when stretched [122]. Other polymers can also be used for fabricating stretchable OFETs, notably P(g2T-T) and P(gT2), both capable of transporting charges at more than 0.35 cm^2^V^−1^s^−1^ through highly aligned crystalline structures interconnected with tie chains and fabricated using TA [123]. Additional information on charge mobilities recently reported for semiconducting polymers based on DPP can be found elsewhere [124].

### 3.2. Carrier Mobility in Thiophene-Based Nanostructures

Possibly the second most studied class of semiconducting polymers in terms of charge mobility—and thus widely employed in OFETs—is the class of polythiophene-based systems. The remarkable charge mobility journey of polythiophenes began in 1986, when polythiophene was shown to exhibit mobilities in the range of 10^−5^ cm^2^V^−1^s^−1^ [125]. This progress has continued to the present day, with maximum mobilities in thiophene-based materials demonstrated to exceed 92 cm^2^V^−1^s^−1^ [96]. Although numerous studies have been reported on common polythiophene systems such as P3OT [126,127] and P3HT [126], the most notable charge mobilities reported in the literature remain the values of 0.62 cm^2^V^−1^s^−1^ [126] and 0.5 cm^2^V^−1^s^−1^ [90], measured in single crystals using OFET and C-AFM configurations, respectively. These values were only surpassed in random polythiophene copolymers, where mobilities of 1.37 cm^2^V^−1^s^−1^ were recorded [128], or in the case of polythiophene isomers, with a reported mobility of 4.6 cm^2^V^−1^s^−1^ [129]. It is important to note that single crystals are highly ordered structures in which all constituent molecules adopt a unique molecular arrangement, with chains often exhibiting a fully extended conformation and end-on orientation [28], which particularly favors intrachain charge mobility. In such molecular assemblies, the side chains may undergo interdigitation, while the backbones interact strongly in the π–π stacking direction leading to small π–π spacings (of only 0.330 nm [28]).

A good mobility of 0.116 cm^2^V^−1^s^−1^ was recently reported in P3HT nanofibers obtained by TA [130]. In this case, a π–π spacing of 0.384 nm was measured. In contrast, nanofibers of P3HT-*b*-PDL displayed a lower mobility of 0.088 cm^2^V^−1^s^−1^, most likely due to weaker stacking along the π–π direction, as indicated by the slightly larger π–π spacing of 0.385 nm [130]. It is also worth mentioning that a slightly higher mobility of 0.12 cm^2^V^−1^s^−1^ was recently measured in regio-regular P3HT domains that were unidirectionally aligned using a unidirectionally floating-film transfer method [131].

In parallel with P3OT and P3HT systems, numerous other thiophene-based systems have been designed and developed, often incorporating BTBT, DNTT, DBTTT, CDT or IDT. Until 2020, such polymers, often aligned with respect to the substrate, exhibited charge carrier mobilities in the range of 10-20 cm^2^V^−1^s^−1^ [107,132,133]. For example, the alignment of CDT-BTZ polymer chains generated hole mobilities of 11.4 cm^2^V^−1^s^−1^ [132], while PDFDSe highly crystalline chains adopting well-packed edge-on orientations were capable of transporting holes at a maximum mobility of 20.3 cm^2^V^−1^s^−1^ [133]. At the same time, electron mobilities in dithiophene-based polymeric systems were shown to be less than 3.8 cm^2^V^−1^s^−1^ [134]. Intriguingly, CDT-BTZ polymer chains assembled into highly ordered fiber-like single crystals led to a mobility of only 5.5 cm^2^V^−1^s^−1^ along the polymer backbone direction [135].

In the past five years, although the record charge mobility values exhibited by this category of thiophene-based materials have not been surpassed, several notable studies have been reported. Notably, these include the fabrication of continuous nanostructures of a D-A PBDP-F2 [136], generation of fibrillar nanostructures of F8T2 [137]; and the realization of nanostructures composed of large crystallite grains of BDT)-based polymer chains [138]. These were achieved using processing techniques such as simple TA, as well as on-center and off-center spin coating combined with TA; these nanostructures exhibited maximum hole OFET mobilities ranging from 1.93 cm^2^V^−1^s^−1^, to 5.4 cm^2^V^−1^s^−1^, to over 8 cm^2^V^−1^s^−1^, respectively. The highest value was recorded for off-center spin-coated structured films displaying a short π–π spacing of 0.382 nm. In contrast, the counterpart film fabricated via on-center spin coating was less densely packed in the π–π direction (0.386 nm) and generated a hole mobility of only 5.56 cm^2^V^−1^s^−1^ [138], demonstrating that a decrease in π–π spacing typically triggers an increase in charge mobility. In conclusion, all the aforementioned charge mobilities are surpassed by the record-high charge mobility of 92.64 cm^2^V^−1^s^−1^ measured in nanowires of PCDTPT (M_w_ = 76 kg/mol) fabricated using liquid-bridge-mediated nano-transfer molding [96].

### 3.3. Carrier Mobility in Naphthalenediimide-Based Nanostructures

NDI-based polymers represent a promising category of semiconducting polymers capable of experiencing high charge mobilities. This subsection begins by revisiting research from approximately ten years ago, when electron mobilities exceeding 1 cm^2^V^−1^s^−1^ were reported for OFETs fabricated from P(NDI2SiC6-T2) [139] and PNDI-RO [140]. Both polymers demonstrated excellent crystallinity and were processed via spin coating followed by TA, resulting in either fibrillar structures with an increased fraction of face-on lamellar orientations [139], or interconnected crystalline grains approximately 120 nm in size with strengthened intermolecular interactions that promote superior charge-transport properties [140].

During approximately the same period, other new NDI-based polymers were reported to display electron mobilities exceeding 5 cm^2^V^−1^s^−1^. For example, the P(NDI2OD-T2) n-type system was pre-aggregated in mesitylene and wire-bar-coated at room temperature to produce shear-aligned nanostructured films with a highly oriented functional surface capable of transporting electrons along the deposition direction at a rate of 6.4 cm^2^V^−1^s^−1^ [141] (note that the same system, but with approximately three times higher molecular weight, was also used to fabricate microwire-like crystals via controlled self-seeding; however, the reported charge carrier mobility only slightly exceeded 2.5 cm^2^V^−1^s^−1^, see ref. [142]). Similar electron mobility values of 6.50 cm^2^V^−1^s^−1^ and 5.64 cm^2^V^−1^s^−1^ were reported for PNDIF-T2 and PNDIF-TVT, respectively [143]. The corresponding thin films, made by spin coating and subjected to TA, consisted of long-range superstructures composed of “backbone” and “side-chain” crystals generated by the strong self-organization of semi-fluoroalkyl segments into highly ordered crystallites [143].

In 2019, Wang and coworkers surpassed the barrier of 7 cm^2^V^−1^s^−1^ for the electron mobility of NDI-based polymers [144]. Specifically, to achieve a highly planar backbone conformation, they incorporated vinylene spacers into an NDI-based polymer, previously synthesized by copolymerizing dibromo-NDI with tin monomers of SNT. Subsequently, they fabricated thin films with enhanced crystallinity and molecular packing, characterized by an exceptionally short π–π stacking distance (0.340 nm) and bimodal distributions of both parallel and perpendicular orientations relative to the π-stacking planes [144]. As a result, a unipolar maximum μ_e_ of 7.37 cm^2^V^−1^s^−1^ was demonstrated [144], a value that remains representative for this class of semiconducting polymers today.

More recently, new NDI-based systems have been reported, although their charge mobilities remain relatively average at present. One notable example is the P(NDI2OD-Se-Th 1.0). Synthesized by Lee and coworkers, this polymer was used to fabricate, via TA, surfaces composed of face-on packed crystalline structures that exhibited a modest OFET charge mobility [145].

### 3.4. Carrier Mobility in Isoindigo-Based Nanostructures

IID-based semiconducting polymers demonstrated significant charge mobility characteristics in 2014, when Kim and coworkers reported a maximum hole mobility of 14.4 cm^2^V^−1^s^−1^ [95] (prior to this, reported charge mobilities were typically in the range of a few cm^2^V^−1^s^−1^, as indicated by refs. [109,110,146]). To achieve this record value, they synthesized a novel D-A polymer named PTIIG-Np and used it to fabricate thin films, which were subsequently exposed to TA to generate highly aligned nanofibrillar networks. These densely ordered lamellar structures, characterized by short π–π stacking distances (0.361 nm) and exhibiting a bimodal distribution of both edge-on and face-on orientations, were responsible for the reported high mobility [95].

Many scientific articles published on IID-based polymers by various research groups between 2015 and 2021 have reported hole mobilities ranging from several to slightly over 8 cm^2^V^−1^s^−1^ [109,110,146,147,148], with a record electron mobility of 9.7 cm^2^V^−1^s^−1^ [149]. The latter was measured in OFET devices incorporating thin films of a highly crystalline polymer (P2FIID-2FBT; based on IID and 3,3′-difluoro-2,2′-bithiophene/2FBT), which, upon TA, formed uniform, large fiber-like polycrystalline grains characterized by effective π–π stacking [149].

More recently, novel IID-based polymers, P(TzII-dTh-dTh) and P(TzII-dTh-dTz), were synthesized by copolymerizing TzII with bithiophene and bithiazole, respectively [150]. Thin films fabricated from these polymers and processed via TA were shown to exhibit poorly crystalline structures. When incorporated into OFET devices, the P(TzII-dTh-dTh) polymer demonstrated the highest hole mobility, while the P(TzII-dTh-dTz) polymer showed the highest electron mobility [150]. However, both values remain modest compared to the other mobility values reported for IID-based polymers discussed above.

### 3.5. Carrier Mobility in Nanostructures Based on Other Semiconducting Polymeric Systems

Highly ordered structures, such as nanowire single crystals composed of rigid rod-like PPE-based polymers, were shown already in 2009 to display good OFET charge carrier mobilities (0.1 cm^2^V^−1^s^−1^) [31]. A year earlier, several polymers —including PFO and PFO copolymers Y80F8:20F5 and S50F8:50F5, which contained, respectively, 20% and 50% 9,9-di(2-methyl)butyl substituted fluorene units, respectively—were aligned into nematic structures by spin coating from heated solutions. However, the resulting time-of-flight charge mobilities were very modest (µ = 2.7 × 10^−4^ cm^2^V^−1^s^−1^) [105].

More recently, Chen and coworkers synthesized new semiconducting PNBDO-FDTE containing varying percentages of additional TFE segments [151]. By spin-casting thin films of these polymers and subjecting them to TA, they generated crystalline structures composed of molecular self-assemblies exhibiting π–π spacings ranging from 0.329 nm to 0.357 nm. Polymers with the highest TFE content demonstrated the highest OFET electron mobilities (µ_e_ = 7.43 cm^2^V^−1^s^−1^, respectively) [151].

**Figure 5:**
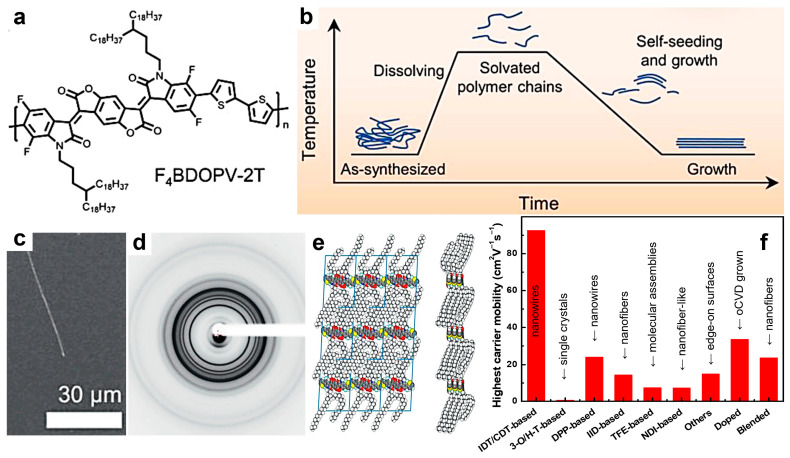
(**a**) Chemical structure of F4BDOPV-2T. (**b**) Diagram depicting the self-seeding method used to grow microwire-like crystals of F4BDOPV-2T. (**c**,**d**) Scanning electron micrograph (**c**) and 2D powder XRD pattern (**d**) of F4BDOPV-2T crystals grown from polymer seeds initially produced by slowly cooling the solution from 140 °C to 25 °C. (**e**) Lamellar packing (left) and π–π packing (right) of F4BDOPV-2T molecules within the microwire-like crystals, as inferred from molecular modeling. Here, gray stands for C, white represents H, red is O, blue is N, cyan is F, and yellow is S. (**f**) Illustration of highest carrier mobilities corresponding to different classes of semiconducting polymers. Reproduced from ref. [142], [Copyright (2021) WILEY-VCH Verlag GmbH with permission from John Wiley and Sons].

Other categories of promising semiconducting polymers exhibiting good charge mobilities include F4BDOPV-2T and polymers based on quinacridone (QA). The highest hole mobility in QA-based systems (slightly over 1 cm^2^V^−1^s^−1^) was demonstrated in hydrogen-bonded self-assembled block- and fiber-shaped structures generated by TA [152]. A relatively high charge carrier mobility was also recently reported in ordered structures of F4BDOPV-2T (Figure 5a), fabricated using the self-seeding method (Figure 5b). These structures were shown to be microwire-like crystals (Figure 5c) characterized by high molecular order (Figure 5d,e) [142]. Due to a lower hopping energy barrier present in such microwire-like structures, a charge carrier mobility of 5.58 cm^2^V^−1^s^−1^ was measured along the polymer chain backbone. Nonetheless, the record electron mobility for this class of polymers remains that reported in 2016 by Zheng and coworkers (14.9 cm^2^V^−1^s^−1^) [153], who fabricated thin films of F_4_BDOPV-2T by combining slow spin coating and TA to generate surfaces composed of large, highly ordered edge-on packed crystallites exhibiting very short π–π stacking distances (0.352 nm) and increased coherence length [153]. A comprehensive comparison between the highest carrier mobilities measured for the aforementioned and other classes of semiconducting polymers can be seen in Figure 5f.

### 3.6. Improved Carrier Mobility in Semiconducting Polymers Blended with Other Polymers

Despite their widespread use in various energy-based applications, semiconducting polymers face significant challenges in future optoelectronic devices, particularly due to their limited environmental stability, poor stretchability, and generally low charge carrier mobility. These limitations are largely influenced by the molecular arrangements of polymer chains at the nano- and microscale. One potential strategy to address these issues is blending semiconducting polymers with insulating and thermoplastic polymers or elastomers. By understanding and controlling processes such as microphase separation in more or less miscible polymer systems, it may be possible to create microstructures that enhance charge transport mechanisms and introduce new material properties, including improved flexibility and stretchability—features that are crucial for the advancement of wearable optoelectronics.

Obviously, one of the most widely used semiconducting polymers in such blends is the P3HT system. Reports in the literature have demonstrated that P3HT can be successfully blended with PDMS, PMMA, PS, PE, HDPE, MEH-PPV, SEBS and others [97]. The most efficient combinations in terms of charge mobility appear to be P3HT/PDMS [154] and P3HT/SEBS [155] blends. The latter exhibit top insulator and bottom semiconductor nanofiber phase-separated morphologies. The P3HT/PDMS microstructure was obtained through solute-solute and solute-surface interactions combined with semiconductor aggregation and exhibited a charge mobility of 0.24 cm^2^V^−1^s^−1^, approximately twice that of analogous neat P3HT films [154]. In contrast, the P3HT/SEBS microstructure was generated through solute-solvent and solute-substrate interactions and demonstrated charge mobilities approaching 0.7 cm^2^V^−1^s^−1^ [155]. However, none of these results match the performance of P3HT nanofibers produced by electrospinning and embedded in a gel composed of PEGDA, HOMPP, and EMIM TFSI, all deposited on stretchable fibers of SBS. This complex system yielded stretchable OFETs capable of charge transport at a record 23 cm^2^V^−1^s^−1^ when unstretched, and 18 cm^2^V^−1^s^−1^ after more than 1500 cycles of stretching at a strain level of ε = 0.7 [156].

Other promising blends (Table 1) include combinations of (i) PDPP-DTT, PDPP3T, PCDTPT, or P(NDI2OD-T2) with PS; (ii) PQT or PDPP-TVT with PMMA; and (iii) PDPP-DTT with SEBS [97]. In these cases, the highest charge mobility of 23.7 cm^2^V^−1^s^−1^ was demonstrated for the PCDTPT/PS microphase-separated morphology, which consists of unidirectional PCDTPT fibrils embedded in the insulating PS matrix. This morphology was obtained through solute–substrate interactions in nanogroove surface relief patterns [157].

More recently, new blend microstructures (Table 1) have been developed, consisting either of semiconducting nanopillars self-assembled under nanoconfinement in a blend of P3HT and PTB7-th [158], or of fibrillar nanostructures and nanoclusters generated by thermally annealing a blend composed of a DPPF-NTz polymer and SEBS [159]. However, the extracted charge carrier mobilities were modest, measuring only 0.73 cm^2^V^−1^s^−1^ and 0.0024 cm^2^V^−1^s^−1^, respectively.

It is also worth mentioning three other interesting blends (Table 1). The first blend consists of P(DPP-T) and BPE [160]. In this case, the microphase-separated granular nanostructures obtained via slot-die coating and direct-ink writing techniques resulted in a hole mobility of only 7 × 10^−4^ cm^2^V^−1^s^−1^ [160]. The second blend is formed by mixing a stretchable PU(DPP)_35_ with PDPPT3_25%_ [161]. This blend, upon TA, generated upon TA large microphase-separated domains exhibiting a charge mobility of 1.28 cm^2^V^−1^s^−1^ [161]. The third blend is a mixture of P2TDPP2TFT4 and SEBS [162]. When subjected to a solution shearing method, this blend formed nanofibers displaying charge mobilities up to 2 cm^2^V^−1^s^−1^ [162].

### 3.7. Enhancing Carrier Mobility of Semiconducting Polymers by Chemical Doping

Unlike inorganic semiconductors, where trace impurities at parts-per-million levels can effectively reduce the band gap, doping semiconducting polymers requires much higher concentrations. Efficient doping of these polymers involves adding strong oxidizing agents for p-type doping or strong reducing agents for n-type doping. This process often affects a substantial portion of the monomer units—typically between one-third and two-thirds—corresponding to doping concentrations of approximately 10^19^ to 10^21^ cm^−3^ [86]. Although chemical or electrochemical doping is primarily used to enhance the electrical performance of semiconducting polymers by either removing an electron from the HOMO through oxidation or adding an electron to the LUMO through reduction, it also has significant consequences on the corresponding charge mobilities.

For example, the pristine PEDOT system, which typically exhibits charge mobilities on the order of 10^−4^ cm^2^V^−1^s^−1^ [163], has been shown to achieve Hall charge mobilities as high as 33.6 23 cm^2^V^−1^s^−1^ in doped PEDOT thin films fabricated from the EDOT monomer and FeCl_3_ oxidant using the oCVD method [164]. PEDOT is often combined with PSS in the well-known PEDOT:PSS composite, which exhibits excellent electrical conductivity and is used in various high-tech applications. More information on the charge mobility of PEDOT, PEDOT:PSS and various PEDOT:PSS composites can be found in recent comprehensive reviews published in the literature [86,165].

Another example illustrating the impact of doping on charge mobility is P3HT. As previously noted, the highest charge mobility in P3HT (0.5 cm^2^V^−1^s^−1^) was reported for highly ordered single crystals composed of fully aligned and interdigitated chains [90]. By chemically doping P3HT, this mobility limit can be increased to 0.8 cm^2^V^−1^s^−1^, as demonstrated by Stanfield and coworkers, who doped P3HT with F_4_TCNQ and optimized the casting solvent for the dopant [166]. P3HT can also be doped with other dopants such as DDB [167] and CPE [168], but the extracted charge mobilities are relatively modest.

In conclusion, the highest charge mobility recorded so far for a semiconducting polymer is 92.64 cm^2^V^−1^s^−1^ measured for nanowires of PCDTPT (Table 1). This value aligns with theoretical studies suggesting that the mobility limit for polymeric systems could reach 100 cm^2^V^−1^s^−1^ [107]. However, we do not discuss in this work the ladder-type 2DCPs which have demonstrated charge mobilities as high as 970 cm^2^V^−1^s^−1^, as recently shown for the 2DCP-MPc ladder-type polymer [169].

Finally, it is worth noting that most of the aforementioned processing methods, which produce various highly ordered nanostructures characterized by optimal molecular packing and high carrier mobilities, rely on widely employed film deposition techniques such as solution shearing, bar coating, spin coating, drop casting, and thermal treatments like TA. Techniques such as solution shearing and bar coating offer greater control and are therefore more efficient in producing densely packed nanostructures, while also demonstrating promising potential for scalability. Consequently, these techniques could be employed in large-scale applications. In contrast, spin coating, drop casting, and similar methods tend to produce nanostructures with less efficient packing at the micro- and nanoscale, especially without TA, and exhibit limited scalability due to the restricted size of the polymer films produced. As a result, these methods are more prone to failure or remain limited in practical device applications.

**Table 1:** Summary of the most recent charge mobilities reported in the literature for various semiconducting polymers. Most relevant results are marked in gray.

Polymer System (* = Full Name)	Molecular Weight (kg/mol)	Nanostructure Type	Processing Strategy	π–π Spacing (nm)	Measurement Technique	Charge Mobility (cm^2^V^−1^s^−1^)	Current On/Off Ratio	Threshold Voltage (V)	Ref.
**DPP-based polymers**									
PDTTDPP	-	nanowires	thermal protocols and drop casting	0.370	OFET	µ = 7	-	−15	[111]
DPPBTSPE	M_n_ = 8	nanowire**s**	thermal protocols and drop casting	0.346	OFET	µ_h_ = 24	10^4^	−4	[29]
DPPBTSPE	M_n_ = 68	nanowire**s**	thermal protocols and drop casting	0.372	OFET	µ = 4.15	10^8^	0	[29]
DPP-2T	M_n_ = 74	strongly packed aggregates	TA	0.380	OFET	µ_h_ = 1.33	10^4^	−3	[113]
PDPPT3	M_n_ = 88	high continuity structures with tie chains	TA	0.372	OFET	µ_h_ = 4.9	10^4^–10^5^	−7 to −15	[114]
PDPPy-Se	M_n_ = 409.1	large-scale crystallites	TA	0.362	OFET	µ_e_ = 2.22	-	−	[112]
P(gDPP-TT) P(gDPP-T2) P(gDPP-MeOT2)	- - -	(highly) ordered and crystalline edge-on oriented surfaces	spin coating	0.358 0.354 0.352	OECT	µ = 0.57 µ = 1.55 µ = 0.28	10^5^ 10^5^ 10^4^	−0.54 −0.52 −0.26	[117]
P(TDPP-BT) P(TDPP-TQ) P(TDPP-BBT)	M_n_ = 42 M_n_ = 16.1 M_n_ = 21	fiber-like domains with face-on packing	TA	0.357 0.369 0.369	OFET	µ_e/h_ = 3.83/2.77 µ_e/h_ = 7.76/6.16 µ_e/h_ = 0.35/0.25	- - -	− − −	[118]
TDPP-Se	M_n_ = 21.2 M_n_ = 40.7 M_n_ = 61.3 M_n_ = 73.3 M_n_ = 135.3	1D rod-like nanostructures	bar-coating	0.369 0.370 0.370 0.369 0.370	OFET	µ_h_ = 6.47 µ_h_ = 13.77 µ_h_ = 12.05 µ_h_ = 6.81 µ_h_ = 7.42	10^3^ ÷10^6^ 10^5^ ÷10^7^ 10^5^ ÷10^7^ 10^4^ ÷10^7^ 10^5^ ÷10^7^	−11 ÷ −6 −8 ÷ −3 −7 ÷ 0 −10 ÷ −2 −12 ÷ −6	[119]
NH-FDPP-TT NH-FDPP-BT	M_w_ = 8.69 M_w_ = 5.61	smooth surfaces with nanopinholes	TA	0.390 -	OFET	µ_h_ = 5 × 10^−3^ µ_h_ = 2.2 × 10^−3^	10^5^ 10^5^	−17.5 −25.4	[120]
PTNDP-IDT PNDP-2T	M_w_ = 41.8 M_w_ = 29.2	coplanar π–π stacked structures	TA	0.386 0.366	OFET	µ_e_ = 0.99 µ_e_ = 0.82	6 6	17−21 15−18	[121]
IDTz-DPP-based	M_n_ = 48.5	amorphous structures without long-range order	TA	-	OFET	µ_e_ = 1.3	-	2.6	[116]
PDPP-TT PDPP-TT-PDMS-1k PDPP-TT-PDMS-2.5k PDPP-TT-PDMS-25k	M_n_ = 17.7 M_n_ = 14 M_n_ = 14.6 M_n_ = 37.5	interconnected fiber-like nanostructures	solution shearing and TA	0.369 0.363 0.370 0.365	OFET	µ = 0.7 µ = 0.21 µ = 0.15 µ = 0.1	~10^5^ ~10^6^ ~10^6^ ~10^6^	22.69 ± 8 29.06± 4 25.76 ± 2 17.08 ± 1	[122]
P(g2T-T) P(gT2)	M_n_ = 67.5 M_n_ = 71	aligned crystalline structures with tie-chains	TA	0.356 0.374	OECT	µ = 0.93 µ = 0.38	- -	- -	[123]
***3-octyl and 3-hexyl-thiophene (3-O/H-T)-based polymers***									
P3OT	M_w_ = 120	single crystal	-	-	OFET	µ = 1.54 × 10^−4^	37	7.3	[127]
P3OT	M_w_ = 51.2	single crystal	-	-	OFET	µ = 0.62	-	-	[126]
P3HT	M_w_ = 39.6	single crystal	-	-	OFET	µ = 1.57 × 10^−3^	-	-	[126]
P3HT	M_n_ = 1.332	single crystal	-	-	C-AFM	µ = 0.5	-	8	[90]
P3HT P3HT-b-PDL	M_n_ = 15 M_n_ = 29.6	nanofibers	TA	0.384 0.385	OFET	µ = 0.116 µ = 0.088	3.9 × 10^5^ 4.5 × 10^5^	−2.6 −2.6	[130]
RR-P3HT	-	unidirectionally aligned domains	unidirectional floating-film transfer method	-	OFET	µ = 0.12	10^4^	-	[131]
***Bithiophene, IDT, CDT-based polymers***									
CDT-BTZ	M_n_ = 50	nanofibers	-	-	OFET	µ = 5.5	10^6^	−60	[135]
s-BTI2-FT f-BTI2-FT	M_n_ = 19.5 M_n_ = 13.8	crystalline edge-on aggregates	TA	0.360 0.360	OFET	µ_e_ = 0.82 µ_e_ = 1.13	10^6^ 10^6^	19 18	[170]
f-BTI2TEG-T f-BTI2TEG-FT	M_n_ = 6.7 M_n_ = 6.3	fibrillar nanostructure	TA	0.353 0.353	OECT	µ_e_ = 0.044 µ_e_ = 0.034	-	0.68 0.53	[171]
					OFET	µ_e_ = 6.34 × 10^−4^ µ_e_ = 3.67 × 10^−4^	16.8 1.3 × 10^3^	- -	
P(g2T-TT) PgBTTT	M_n_ = 7 M_n_ = 10	2D islands of elongated parallel backbones	drop-casting and electrospray deposition	0.358 0.354	OECT	µ_h_ = 0.41 µ_h_ = 3.44	- -	- -	[172]
PBDP-F2	M_n_ = 40.9	continuous donor and acceptor nanostructures	TA	0.369	OFET	µ_h_ = 1.93	-	-	[136]
F8T2	-	fibrillar structures	TA	-	OFET	µ_h_ = 5.40	>10^5^	-3	[137]
BDT-based	M_n_ = 12.8	nanostructures comprising large crystallite grains	on-center vs. off-center and TA	0.386 0.382 - -	PMMA-gated PMMA-gated pristine OFET functionalized	µ_h_ = 0.44 × 10^−2^ µ_h_ = 1.90 × 10^−2^ µ_h_ = 5.56 µ_h_ = 8.03	>10^2^ >10^2^ >10^5^ ~10^6^	−16.90 −26.64 −1.25 −1.47	[138]
C_16_-IDT-BT	M_w_ = 108	-	TA	0.410	OFET	µ_h_ = 1.2	-	−	[173]
									
IDT-BT TTIF-BT	M_n_ = 58 M_n_ = 51	surfaces of enhanced crystalline order	TA	0.410 0.414	OFET	µ_h_ = 1.5 µ_h_ = 1.1	- 10^6^	0 −12	[174]
									
									
PCDTPT	M_w_ = 76	nanowires	liquid-bridge-mediated nano- transfer molding	0.442	OFET	µ = 92.64	1.8 × 10^4^	1.77	[96]
***IID-based polymers***									
Polyisoindigo Poly(ethynylisoindigo) Poly(bisisoindigo)	M_n_ = 21.9 M_n_ = 15.4 M_n_ = 15.5	surfaces with different molecular order	TA	0.370 0.350 0.380	OFET	µ_e_ = 2.85 × 10^−4^ µ_e_ = 4.15 × 10^−4^ µ_e_ = 7.67 × 10^−5^	10^4^ 10^4^ 10^4^	59 57 51	[175]
P(TzII-dTh-dTh) P(TzII-dTh-dTz)	M_n_ = 236 M_n_ = 138	poorly crystalline structures	TA	- -	OFET	µ_h/e_ = 1.43/0.55 µ_h/e_ = 0.38/0.56	10^3^ ~3	15.96 79.78	[150]
P2FIID-2FBT	M_n_ = 59	fiber-like poly-crystalline grains	TA	0.350	OFET	µ_e_ = 9.7	10^3^–10^4^	57	[149]
PTIIG-Np	M_n_ = 21	nanofibers	TA	0.361	OFET	µ_h_ = 14.4	-	−45÷ −48	[95]
***NDI-based polymers***									
P(NDI2OD-Se-Th 1.0)	M_n_ = 70	surfaces comprising face-on packed crystalline structures	TA	-	OFET SCLC	µ = 0.138 µ_e_ = 2.5 × 10^−4^	>10^3^ -	1.3 -	[145]
P(NDI2OD-T2)	M_n_ = 76.6	microwire			OFET	µ = 2.56	-	-	[142]
SNTandNDI-based	M_w_ = 54.9	nanofiber-like crystalline structures	TA	0.340	OFET	μ_e_ = 7.37	10^6^–10^7^	1-5	[144]
***TFE-based polymers***									
PNBDO-FDTE100 PNBDO-FDTE90 PNBDO-FDTE80 PNBDO-FDTE70 PNBDO-FDTE60 PNBDO-FDTE0	M_w_ = 164.4 M_w_ = 117.6 M_w_ = 115.5 M_w_ = 108.1 M_w_ = 105.5 M_w_ = 76.9	crystalline structures comprising molecular self-assemblies	spin coating and TA	0.350 0.350 0.353 0.356 0.357 0.329	OFET	µ_e_ = 7.43 µ_e_ = 7.25 µ_e_ = 6 µ_e_ = 1.75 µ_e_ = 062 µ_e_ = 0.182	10^2^–10^3^ 10^2^–10^3^ 10^2^–10^3^ ~10^2^ ~10^2^ 10^2^–10^3^	19.18 18.76 17.5 18.4 14.75 17.73	[151]
***Other types of polymers***									
PFO Y80F8:20F5 S50F8:50F5	-	aligned nematic structures	spin coated from heated solutions	-	time-of-flight	µ = 2.7 × 10^−4^ µ = 3.7 × 10^−2^ µ = 2.7 × 10^−2^	-	-	[105]
TA-PPE	M_w_ = 51.328	nanowire			OFET	µ = 0.1	-	−40	[31]
F_4_BDOPV-2T	M_n_ = 60.4	microwire			OFET	µ = 5.58	10^3^-10^4^	2	[142]
F_4_BDOPV-2T	M_n_ = 38	surfaces of edge-on packed crystallites	TA	0.352	OFET	µ_e_ = 14.9	10^3^-10^4^	−17	[153]
QA based	M_w_ = 38.5	block- and fiber-shaped structures	TA	0.356	OFET	µ_h_ = 1.02	5-6	−17	[152]
***Doped polymers***									
P3HT/CPE	M_w_ = 80	ordered crystallites	dopant induced	-	OFET	µ_h_ = 0.135	~10^3^	−22.4 ± 2.1	[168]
P3HT/DDB	M_n_ = 50–70	lamellar crystallites	spin coating	-	AC-Hall effect	µ = 0.095	-	-	[167]
PEDOT/FeCl_3_	-	-	oCVD	-	AC-Hall effect	µ = 33.6	-	-	[164]
***Blended polymers***									
PCDTPT/PS	M_w_ = 50	nanofibers	slow-drying	-	OFET	µ_h_ = 23.7	-	-	[157]
P3HT PTB7-th P3HT/PTB7-th	- - -	nanopillars nanopillars nanopillars	self-assembly under nano confinement	0.373 0.391 0.378:0.387	conductive scanning force microscopy	µ_vertical_ = 0.96 µ_vertical_ = 0.036 µ_vertical_ = 0.73	- - -	- - -	[158]
P2TDPP2TFT4/SEBS	M_n_ = 97	nanofibers	solution shearing	0.366	OFET	µ = 2	10^4^–10^5^	-	[162]
PU(DPP)_35_ PU(DPP)_35_/PDPPT3_25%_	M_n_ = 20 -	tens to hundred nm large phase- separated domains	TA	- 0.364	OFET	µ_h_ = 0.19 µ_h_ = 1.28	- -	- -	[161]
P(DPP-T)/BPE	M_w_ = 146.3	phase-separated granular nanostructures	slot-die coating	-	OFET	µ_h_ = 1.37 × 10^−2^	10^2^	14.6	[160]
			direct-ink writing	-		µ_h_ = 7 × 10^−4^	10^1^	−13.3	
DPPF-NTz/SEBS	M_n_ = 34	fibrillar nanostructures and nanoclusters	TA	0.390	OFET	µ_e_ = 0.0024	10^5^	4.61	[159]
P3HT/PEGDA/HOMPP based	M_wP3HT_ = 87	nanofibers	electrospinning	-	OFET	µ = 23	105	−3÷ −2.7	[156]

At the end of this section, one must emphasize that chemical doping and polymer blending are widely employed strategies to tailor the optoelectronic properties of semiconducting polymers. However, over the long term, these approaches often introduce significant challenges related to the stability and reproducibility of polymer film properties. These challenges include: (i) chemical instability of dopants (or other blend components), where dopant molecules can diffuse out of the polymer matrix, undergo chemical transformations, or degrade under environmental conditions such as humidity or light exposure, leading to a time-dependent loss of doping efficiency; (ii) morphological instability caused by poor miscibility between various components (e.g., polymer–additive, polymer–fullerene, polymer–polymer, polymer–dopant), resulting in phase segregation through crystallization or aggregation—dopants, small acceptor molecules, or additives can crystallize or aggregate into new morphological structures that disrupt carrier transport pathways; (iii) non-uniform doping and dedoping effects, where even in rare cases when homogeneous doping at the nanoscale is achieved, it does not persist due to slow desorption of dopants, which decreases the free carrier concentration; (iv) reproducibility challenges related to variations in polymer synthesis, dopant or additive purity, inconsistent processing conditions (for example, caused by minor changes in solvent or substrate temperature, humidity variations, etc.), or kinetic trapping of blended or doped films in often unreproducible metastable states; (v) optoelectronic trade-offs caused by photoluminescence quenching and broadening or shifting of absorption and emission spectra; and (vi) degradation of device interfaces, typically induced by the migration of dopant ions or additive molecules to various interfaces, which compromises interfacial stability and, consequently, device performance.

## 4. The Enhancement of Electrical Conductivity in Nanostructures of Semiconducting Polymers

Electrical conductivity measures a material’s capability to permit the flow of electric current, indicating how easily charge carriers can move through the material when an electric field is applied. The bulk conductivity of a semiconducting polymer is typically determined by charge transport both along individual polymer chains and between adjacent chains. It depends on the type of charge carriers, their mobility and density [40]. While the latter can be effectively tuned by chemical doping—as discussed in this subsection, doping is a widely used method to enhance electrical conductivity in semiconducting polymers, potentially increasing it from insulating to metallic levels (i.e., from 10^−10^–10^−5^ S/cm to 10^4^ S/cm [176])—the charge transfer process plays a crucial role in enhancing conductivity at the micro- and nanoscale. This process typically occurs from one crystallite to another and across tie chains connecting crystallites [86]. Therefore, highly ordered nanostructures exhibiting strong interchain packing (i.e., decreased π–π stacking distance) generally promote charge delocalization along and across polymer chains through mechanisms such as hopping and intrachain coupling. Additional factors influencing electrical conductivity in semiconducting polymers are comprehensively reviewed in a recent publication by Pooja and coworkers [177].

There are many semiconducting polymers that exhibit good electrical conductivity, with the most common being PEDOT, PANI, PPy, P3HT, PA, and several others (Table 2). Among these, PEDOT stands out as the most prominent, in terms of electrical conductivity and practical applications. When polymerized via a chemical oxidative method in an aqueous solvent using Fe_2_(SO_4_)_3_ as the oxidant, PEDOT shows a conductivity in the range of 0.0002–0.001 S/cm [178]. This conductivity can be significantly increased to 130 S/cm by assembling PEDOT molecules into high aspect-ratio nanofibers through evaporative vapor-phase polymerization inside a CVD chamber, followed by TA [179]. PEDOT’s conductivity can be further enhanced by employing a novel synthetic strategy that induces surfactant-free interfacial polymerization at the interface of PEDOT and flexible cellulose paper. The resulting PEDOT paper, composed of densely packed π-conjugated chains, exhibits a conductivity of approximately 375 S/cm [180]. Moreover, Massonnet and coworkers synthesized PEDOT-based polymers displaying metallic-like behavior, by using Fe(OTf)_3_ as the oxidant, achieving a conductivity of 1218 S/cm for PEDOT:OTf [181]. Subsequent treatment of the PEDOT-based polymer in TsOH, TfOH and H_2_SO_4_ further enhanced its electrical conductivity to 1503 S/cm, 1613 S/cm, and 2273 S/cm, respectively [181]. These high conductivity values for PEDOT were surpassed by molecularly engineering the crystallization and morphology of PEDOT using oCVD, followed by treatment with hydrobromic acid and precise control of film thickness [182]. The resulting films, composed of highly crystalline, face-on oriented PEDOT chains, exhibited a record-high conductivity of 6259 S/cm [182].

For many technological applications, it is useful to blend PEDOT with PSS to generate PEDOT:PSS water-soluble hole transport layers. Due to the non-conductive nature of PSS, the PEDOT:PSS system exhibits a relatively low conductivity of around 0.02 S/cm [183], but it can typically reach values between 0.25 S/cm [184] and approximately 1 S/cm [181]). Guo and coworkers demonstrated that PSS can be replaced by poly(diphenylamine-4-sulfonic acid) (PDAS) to create a novel water-soluble hole transport layer with significantly higher conductivity (0.135 S/cm) [183]. Fortunately, many formulations of PEDOT:PSS that can be further enhanced with various additives to improve conductivity [185]. Until 2015, the best formulation appeared to be the commercial PEDOT:PSS known as Clevios P, to which DMSO was added. This treatment increased the electrical conductivity from 0.2 to 1 S/cm to 677 S/cm (many other relevant formulations are documented elsewhere [185]). By 2019, these formulations had been further optimized, achieving conductivities ranging from a few thousand to several thousand S/cm [186]. Two of the highest conductivities were obtained for PEDOT:PSS commercially known as PH1000, either by depositing it as a transparent conductor film using the solution shearing approach [187] or by mixing it with additives followed by light oxygen plasma treatment [188]. Consequently, a conductivity of 4600 S/cm was measured in the former case [187], while a record-high value of 5012 S/cm was recorded in the latter [188].

**Figure 6:**
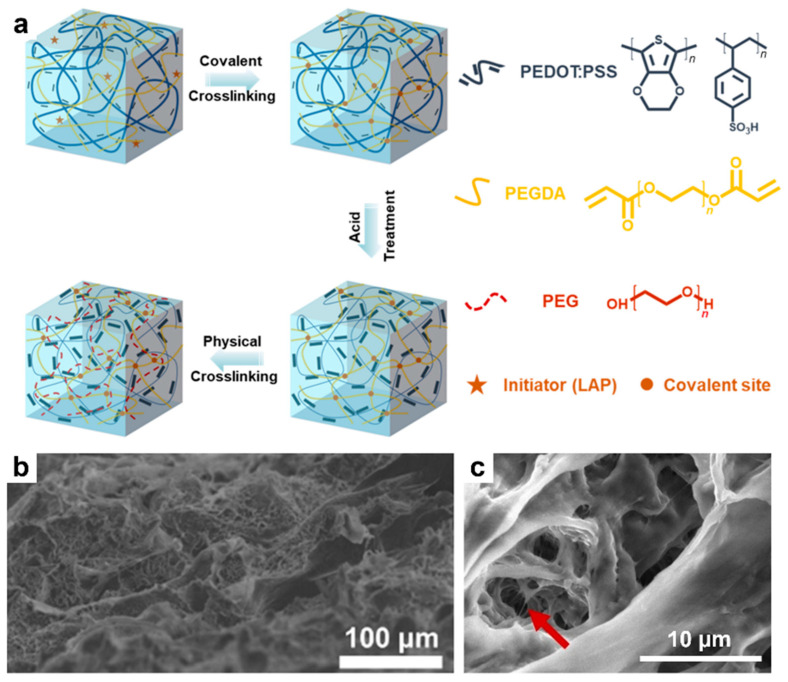
(**a**) Diagram depicting the triple-network strategy used to prepare the TN–H hydrogel. (**b**) A cross-sectional scanning electron microscopy (SEM) image depicting the TN–H gel. (**c**) A SEM micrograph of the TN-H freeze-dried hydrogel emphasizing the interconnected fibers in its pores (red arrow). Reproduced from ref. [189], [Copyright (2023) the authors, SmartMat published by Tianjin University and John Wiley and Sons Australia, Ltd., with permission from John Wiley and Sons].

More recently, Chen and coworkers employed a triple-network strategy (Figure 6a) by incorporating a covalently crosslinked network of PEGDA into a PEDOT:PSS hydrogel doped with DMSO. The resulting three-dimensional (3D) porous system was then soaked in acetic acid and rinsed with water. This process was followed by the diffusion of additional PEG chains into the porous structure, yielding a stretchable triple-network hydrogel (TN-H) composed of phase-separated PEDOT and PSS domains (Figure 6b) and pores containing interconnected fibers (Figure 6c). The TN-H system exhibited an overall electrical conductivity of 0.3 S/cm at a stretchability of 900%, a benchmark value to date for all stretchable PEDOT:PSS-based gels [189].

Another class of polymers displaying highly effective conductive properties includes PPy and DPP-based systems. Electrical conductivity ranges from 10^−6^ S/cm for neat DPP-based films [190,191], to 12 S/cm for PPy hydrogels [192], and up to 70 S/cm for DPP-based polymers doped withCN6-CP [190]. Doping DPP-based polymers with FeCl_3_ has been shown to further increase conductivity from the range of 19–119 S/cm [191,193] to a staggering 997 S/cm [194]. In contrast, the use of N-DMBI dopant typically results in more modest conductivities, ranging from 0.0001 to 8.4 S/cm [124].

**Figure 7:**
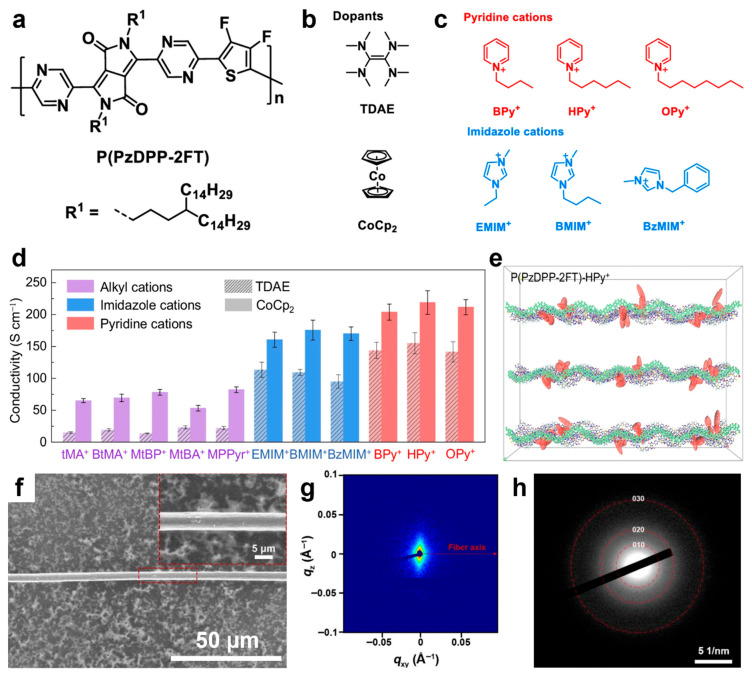
(**a**–**c**) Schematics depicting the P(PzDPP-2FT) (**a**), TDAE) and CoCp_2_ dopants (**b**) and pyridine and imidazole aromatic cations (**c**). (**d**) Plot juxtaposing the electrical conductivities recorded for doped P(PzDPP-2FT) films that were docked with various cations. (**e**) A molecular dynamic simulation emphasizing the docking of P(PzDPP-2FT) with HPy^+^ cations. (**f**–**h**) SEM image (**f**), 2D small-angle x-ray scattering (SAXS) pattern (**g**) and selected area electron diffraction (SAED) of a P(PzDPP-2FT) fiber. Reproduced from ref. [201] (**a**–**e**) [Copyright (2024) The author(s), with permission from Springer Nature] and ref. [98] (**f**–**h**) [Copyright (2024) the authors, some rights reserved; exclusive licensee American Association for the Advancement of Science].

Recently, novel DPP-based polymers have been reported in the literature, including PDPIN [195], PTz-5-DPP [196], P(DPP-CNPz) [197], as well as P(DPP-DCNPz) [197]. The PTz-5-DPP, P(DPP-CNPz), and P(DPP-DCNPz) systems doped with N-DMBI delivered conductivities of 8.31 S/cm, 25.3 S/cm, and 33.93 S/cm, respectively. In contrast, the PDPIN system doped with PSpF demonstrated a higher conductivity of 78.1 S/cm. This promising value has been challenging to surpass, even in more recent DPP-based polymers doped with N-DMBI, such as the ThDPP-CNBTz system (56.5 S/cm) [198]; the DPP-BFDO-Th system, based on a quinoidal BFDO unit (65.68 S/cm) [199]; and the PDFSe system, based on an acceptor-triad moiety of DFSe (62.6 S/cm) [200]. The only higher conductivity values—218 S/cm and up to 170 S/cm —were recorded for P(PzDPP-2FT) (Figure 7a) doped with TDAE or CoCp_2_ (Figure 7b) and further docked with pyridine and imidazole aromatic cations (Figure 7c), respectively [201]. Other significant conductivities in the range of 50–100 S/cm were also measured for P(PzDPP-2FT) docked with various alkyl cations (Figure 7d). These high electrical conductivities were attributed to decreased energetic disorder in the P(PzDPP-2FT)-based system, resulting from docking the cations near the polymer backbone (Figure 7e) [201]. A similar conductivity of 173 S/cm was reported for n-doped P(PzDPP-2FT) fibers (Figure 7f) fabricated using a flow-enhanced crystallization method based on shear flow-induced disaggregation and alignment. These fibers comprised highly planar backbones and crystallized alkyl side chains, which promoted strong interchain π–π stacking interactions (Figure 7g,h) that enhanced electrical conductivity [98]. Additional conductivity data for other polymers doped with various chemicals can be found elsewhere [177].

Neat films of P3HT, as well as other thiophene-based systems—including PBTTT derivatives—typically display low electrical conductivities in the range of 10^−6^ S/cm [202,203]. Upon optimal doping with F_4_TCNQ, the conductivity of these polymeric systems increases dramatically to 1-2 S/cm [202,203,204], with more recent reports demonstrating conductivities reaching up to 5.6 S/cm [166]. Interestingly, doping aligned films of P3HT and PBTTT derivatives with FeCl_3_ has yielded exceptional conductivities of 570 S/cm and 2.2 × 10^5^ S/cm, respectively [205]. Additional conductivity values ranging from 3 × 10^−4^ to 251 S/cm have been reported for P3HT (including both regioregular and regiorandom forms) doped with various other molecules. These findings are comprehensively summarized in a recent dedicated study [206]. Similarly, notable combinations of PBTTT with various dopants, exhibiting conductivities in the range of 36-1300 S/cm, are documented elsewhere [99].

**Figure 8:**
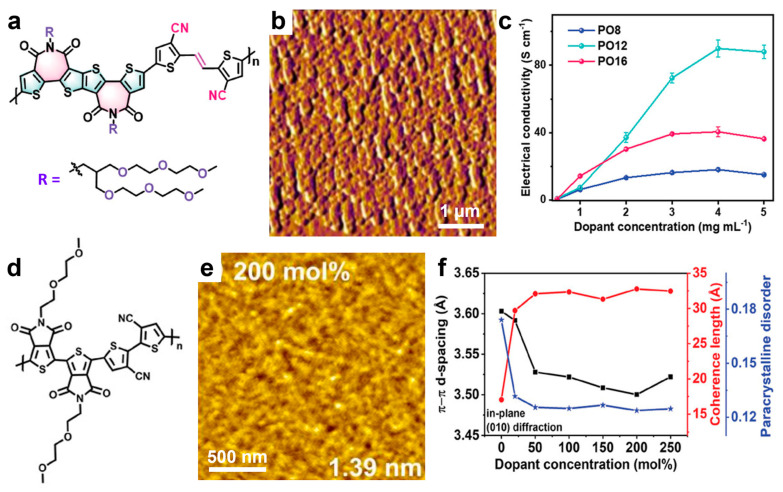
(**a**) Chemical structure of PO12 system. (**b**) AFM phase image depicting the morphology of a PO12 film n-doped with N-DMBI. (**c**) Electrical conductivities measured against the dopant concentration for PO12 and its analogs displaying longer and shorter side chains, respectively. (**d**) Chemical structure of n-PT4. (**e**) AFM topography image depicting the morphology of a doped n-PT4 films using a dopant concentration of 200 mol%. The rms surface roughness is shown in the bottom-right corner. (**f**) The dependance of *π*-*π* stacking distance, coherence length, and paracrystalline disorder on the doping concentration in n-PT4 films. Reproduced from ref. [208] (**a**–**c**) [Copyright (2023) the authors. Advanced Science published by Wiley-VCH GmbH, with permission from John Wiley and Sons], and ref. [207] (**d**–**f**) [Copyright (2023) Wiley-VCH GmbH, with permission from John Wiley and Sons].

Other recently developed thiophene-based systems reported to display promising electrical conductivity include polymers such as n-PT4, [207]PO12, [208]*,* PCNI2-BTI polymer [209], and f-BSeI2TEG-FT [210]. For example, the PO12 system (schematically depicted in Figure 8a) doped with N-DMBI, was shown to generate films composed of large, highly crystalline domains intercalated with small dopant domains (Figure 8b) and delivered a conductivity of 92 S/cm at a dopant concentration of 4 mg/mL (Figure 8c) [208]. Notably, analogous systems based on longer or shorter side chains exhibited significantly lower conductivities. Higher conductivities of 103.5 S/cm [210] and 133.3 S/cm [207] were achieved for the f-BSeI2TEG-FT and n-PT4 systems, respectively, when doped with N-DMBI. In the latter case, due to the extremely short side chains of n-PT4 (Figure 8d), doping induced a crystalline fibril-textured morphology (Figure 8e) with improved molecular order, characterized by a short *π*–*π* stacking distance of only 0.350 nm (compared to 0.360 nm before doping; see Figure 8f) [207]. Moreover, this efficient *π*–*π* stacking of highly doped n-PT4 chains was accompanied by an increased coherence length in n-PT4 films and decreased paracrystalline disorder (Figure 8f). These observations demonstrate that high doping efficiency can be achieved without introducing additional structural disorder into the film microstructure, thereby greatly favoring the increase in conductivity.

A higher conductivity of 150.2 S/cm was measured for thin films of PCNI2-BTI doped with N-DMBI [209]. These films were prepared via solution shearing, as spin coating proved less efficient, and consisted of highly oriented, fiber-like aggregates showing a preferential face-on orientation. It is worth noting that other imide-based polymeric systems, such as the PDTzSI-Se have demonstrated a conductivity of 164 S/cm when doped with N-DMBI [211]. analogs with lower selenophene content, doped similarly, demonstrated conductivities in the range of 62–98 S/cm.

Other notable polymeric systems exhibiting high electrical conductivity include those based on PPV. Among these are the FBDPPV and FBDPPV-OEG systems [212,213]. When doped with a TAM derivative, thin films of FBDPPV demonstrated conductivities up to 21 S/cm [213]. This value increased slightly to 22.5 S/cm by using shorter FBDPPV chains [212]. In contrast, the FBDPPV-OEG/TAM doped system, initially dissolved in hexafluoroisopropanol, formed disconnected porous regions that facilitated dopant penetration, resulting in a higher conductivity of 39 S/cm [214]. A comparable conductivity (38.3 S/cm) was also observed in films of the PFClTVT system, doped with N-DMBI and composed of nanofiber-like aggregates [215].

Another system worth mentioning in this section is oligoaniline TANI, which can be processed from solutions to generate plate-like single crystals vertically aligned with respect to the graphene guiding substrate. These crystals were shown, using C-AFM, to possess an electrical conductivity of 12.3 S/cm along the π-stacking direction [216]. This value exceeds by more than an order of magnitude the highest conductivity previously reported for TANI in wire-, ribbon-, or plate-like structures produced using a solvent exchange method [217]. It also surpasses the typical conductivity values of 0.1 to 1 S/cm recorded for PANI [176,218], although PANI is capable of reaching conductivities up to 200 S/cm [219]. Moreover, even higher conductivity values of 320 S/cm have been obtained for PANI when self-assembled into tubes with a diameter of 140 nm and doped with TSA [220]. Additional relevant conductivity values for doped PANI can be found elsewhere [177].

Furthermore, polymers based on NDI and doped with N-DMBI have been shown to exhibit moderate electrical conductivities, ranging from 0.008 S/cm for P(NDI2OD-T2) (or N2200) to 1.8 s/cm for the PNDI2TEG-2Tz system [124]. Additional polymer/dopant configurations and their corresponding conductivities can be found in comprehensive recent reviews [99,221,222].

Other polymeric systems exhibiting promising electrical conductivities include biodegradable materials based on PANI, PEDOT, or PPy. Detailed information on the generated nanostructures and their corresponding electrical conductivities can be found elsewhere [223]. Additionally, it is worth mentioning the report by Matsuhisa and coworkers, who prepared an elastic conductor ink by mixing Ag flakes with an elastomeric fluorine copolymer and a water-based fluorine surfactant in an organic solvent [224]. This process resulted in a phase-separated morphology consisting of an elastic core covered by an Ag-dense surface layer, which exhibited an initial conductivity of 738 S/cm. This value decreased to a still high conductivity of 182 S/cm when the elastic conductor was stretched to 215% strain [224]. In this work, we have again excluded ladder-type polymers, which can achieve conductivities in the range of few thousand S/cm. An example of such a system is PBFDO, which can exhibit conductivities exceeding 2000 S/cm in films composed of molecules adopting a preferential edge-on orientation [225].

Finally, it is worth noting that two primary trade-offs remain unresolved or only partially addressed in organic optoelectronics. The first trade-off involves balancing charge carrier mobility with the thermal and morphological stability of thin films composed of semiconducting polymers. This challenge arises from conflicting structural and chemical requirements: achieving high mobility demands high crystallinity and well-ordered, efficiently π–π-stacked nanostructures, whereas such crystalline nanostructures are thermodynamically less stable due to phase segregation caused by possible reorientation, coarsening, or further growth. Therefore, a critical challenge is to identify optimal solutions that maintain high crystallinity for morphological stability without significantly compromising device performance. This entails designing polymers that form nanostructures with a high degree of local order while suppressing macroscopic phase changes through crosslinking or non-covalent interactions. The second trade-off concerns the balance between flexibility/stretchability and electrical conductivity in thin films of semiconducting polymers, which is crucial for advancing flexible optoelectronics, wearable devices, and organic circuits. Electrical conductivity requires long conjugation lengths in planar, rigid backbones, whereas increased flexibility demands deformable backbones composed of soft, potentially elastomeric segments. Moreover, although high crystallinity and doping improve π–π stacking and charge transport, highly crystalline and doped structures are prone to cracking under mechanical strain. Potential solutions include developing semiconducting block copolymers that incorporate elastomeric blocks to combine conductive and stretchable domains; semiconducting polymer-based composites that maintain good conductivity while remaining highly flexible; or ductile crystalline polymers that preserve order under mechanical strain.

**Table 2:** Summary of conductivity values recently reported in the literature for various semiconducting polymers. Most relevant values are marked in gray.

Polymer System/Dopant (If the Case)	Molecular Weight (kg/mol)	Nanostructure Type	Processing Strategy	Measurement Configuration	Electrical Conductivity (S/cm)	Ref.
PEDOT	-	scale-like morphology	chemical oxidative method and TA	four-probe measurements	0.0002–0.001	[178]
PEDOT	-	nanofibers	evaporative vapor-phase polymerization	four-probe measurements	130	[179]
PEDOT	-	microfibers	surfactant-free interfacial polymerization	four-probe measurements	375	[180]
PEDOT	-	face-on fibrillar domains	oCVD and acid treatment	four-probe measurements	6259	[182]
PEDOT:PSS	-	well-defined (elongated) nanofibers	solution shearing, TA, treated with methanol	four-probe measurements	4600	[187]
PEDOT:PSS/ additives	-	fibrous morphology	blending with additives and light oxygen plasma treatment, + soaking in solvents and TA	four-probe measurements	5012	[188]
PEDOT:PSS/PEGDA/PEG	-	phase-separated PEDOT and PSS domains	triple-network strategy: incorporating PEGDA in PEDOT:PSS, diffusion of PEG	four-probe measurements	0.3	[189]
Selenium substituted DPP	-	ordered edge-on oriented crystalline structures	drop casting followed by immersion in dopant solution	four-probe measurements	977	[194]
PDFSe	M_n_ = 95.5	near-amorphous featureless surfaces	spin coating and TA	four-probe measurements	62.6	[200]
PTz-5-DPP/N-DMBI	M_n_ = 22	mixed face-on and edge-on orientated structures	spin coating and TA	four-probe measurements	8.31	[196]
P(PzDPP-2FT)	-	structures of pyridine counterions docked near the polymer backbone	spin coating followed by immersion in dopant solution	four-probe measurements	218	[201]
P3HT/FeCl_3_	M_n_ = 24.2	π-stacked ordered domains of highly aligned polymer chains intercalated with dopants	doctor blading of hot polymer solutions followed by high-temperature film rubbing and TA	four-probe measurements	570	[205]
PBTTT derivative/FeCl_3_	M_n_ = 26	π-stacked ordered domains of highly aligned polymer chains intercalated with dopants	doctor blading of hot polymer solutions followed by high-temperature film rubbing and TA	four-probe measurements	2.2 × 10^5^	[205]
PDTzSI-Se/N-DMBI	M_n_ = 145.7	crystalline structures exhibiting high backbone coplanarity	spin coating and TA	-	164	[211]
PO12/N-DMBI	M_n_ = 23.8	large highly crystalline domains intercalated with small dopant domains	spin coating and TA	-	92	[208]
n-PT4/N-DMBI	M_n_ = 137	crystalline fibril-textured morphology	spin coating and TA	four-probe measurements	133.3	[207]
PCNI2-BTI/N-DMBI	M_n_ = 70.9	oriented-fiber-like aggregates	solution shearing and TA	-	150.2	[209]
FBDPPV-OEG/TAM	M_n_ = 65.63	disconnected regions of pore structures	spin coating followed by vapor doping and TA	four-probe measurements	39	[214]
PFClTVT/N-DMBI	M_n_ = 39.4	nanofiber-like aggregates	spin coating and TA	four-probe measurements	38.3	[215]
TANI	-	single crystals	grown from solution on guiding substrates	C-AFM	12.3	[216]
P3HT/DDB	M_n_ = 50–70	lamellar crystallites	spin casting	AC-Hall effect	12.9	[167]
PANI/TSA	-	self-assembled nanotubes	template-free and interfacial polymerization method	four-probe measurements	320	[220]

## 5. Latest Photovoltaic Properties of Polymer-Based Semiconducting Nanostructures

Various nanostructures of semiconducting polymers, exhibiting different degrees of ordering, are also significantly important in the design and development of novel OSC energy devices. This includes OSCs based on fullerene acceptors, non-fullerene acceptors, as well as all-polymer OSCs. The efficiency of these devices has been shown to strongly correlate with the molecular arrangements adopted by the donor and acceptor molecules in the active layer, at both the nano- and microscale [72,226,227], since internal processes such as charge transfer and transport depend heavily on the film microstructure.

### 5.1. Nanostructures with Enhanced Photovoltaic Properties Found in Fullerene-Based OSCs

Fullerene-based binary OSCs feature an active layer composed of a donor polymer and a fullerene-based acceptor. The ideal morphology in these devices should facilitate efficient charge transfer at the donor-acceptor interface, as well as subsequent charge migration toward the respective electrodes. This morphology typically includes interpenetrating, bi-continuous donor-acceptor networks. An advantage of this type of OSC is the spherical shape of fullerene acceptors, which enhances their ability to accept and transport electrons isotropically with relatively high mobilities [228,229].

Until 2017, fullerene-based OSCs were shown to approach a PCE limit of approximately 12%. This limitation was primarily caused by energy losses due to charge transfer states, as well as radiative and nonradiative recombination within active layers that had insufficiently controlled microstructures [230]. The most extensively studied combinations involved polythiophenes such as P3HT and PCBM or their derivatives (C60, PC71BM, etc.). These bulk heterojunctions achieved PCEs ranging from several percent up to over 10% [228,231]. Even highly ordered P3HT structures—such as needle-like and hairy single crystals and nanofibers of varying molecular weights mixed with PC71BM—did not exceed PCEs of a few percent [232] (Table 3). This outcome is unsurprising, as P3HTs generally exhibit limitations, including a large optical bandgap that restrict their absorption range [229].

A notable study reporting PCEs approaching 12% was conducted by Zhao and coworkers in 2016. They utilized the novel PffBT4T-C_9_C_13_ in combination with PC71BM to fabricate active layers with enhanced microstructure. These films were spin-coated from TMB-PN. Compared to PffBT4T-C_9_C_13_:PC71BM films processed from TBM alone, those processed from the TMB-PN mixture exhibited polymer backbones that were more oriented relative to the substrate and adopted face-on orientations. Moreover, processing from TMB-PN simultaneously reduced domain size and increased domain purity in the PffBT4T-C_9_C_13_:PC71BM films [93]. As a result, the PCE improved from 6.4% to 11.7%. Although more recent studies have employed other donor polymers with PC71BM, such as IID-based [146] and NTz-based [233] systems, the reported PCE values remain in the range of a few percent up to 11% [146,233]. Therefore, to the best of our knowledge, PffBT4T-C_9_C_13_:PC71BM OSCs remain the most efficient polymer:fullerene OSCs fabricated to date. This limitation is most likely due to the poor absorption of fullerenes and the significant voltage loss present in such OSCs [229].

The only polymer:fullerene-based OSCs that significantly surpassed the 12% PCE limit were later achieved by incorporating a non-fullerene acceptor (NFA) alongside the fullerene electron-accepting component in so-called ternary OSCs [231] (sometimes with an additional dopant included [234]). A notable example includes OSCs composed of polythiophenes with cyano-group substitutions and varying degrees of fluorination (P5TCN-F25) mixed with [70]PCBM and the Y6 NFA, achieving a PCE of 17.2% [231]. Another example was reported by Lin and coworkers, who used neutral Diquat (DQ) as an n-dopant to maximize the PCE of ternary PM6:BTP-eC9:PC71BM OSCs to 18.3% [234].

### 5.2. Nanostructures Employed in Efficient OSCs Based on Non-Fullerene Acceptors

To address some of the limitations of polymer:fullerene OSCs, such as poor absorption, limited tunability of fullerene structures, and significant voltage loss, fullerene acceptors have been increasingly replaced by a variety of newly developed NFA small molecules. These NFAs not only efficiently transport electrons but also exhibit reduced voltage loos, thereby enabling higher PCEs in polymer:NFA OSCs. Additionally, NFAs are easier to design and synthesize, and the morphology of the resulting polymer:NFA blends can be tuned and optimized more straightforwardly [229].

Until 2017, the most efficient polymer:NFA OSCs were those based on wide:low, low:low, and medium:medium bandgap blends, achieving PCEs of approximately 13%, 12%, and over 10%, respectively [230]. Other polymer:NFA blends involving low:wide and wide:wide bandgap systems resulted in OSCs with PCEs ranging from a few percent to less than 10% [230]. Most NFAs were based on the perylene diimide (PDI) core unit, known for its high electron mobility and deep LUMO level, which is compatible with most polymers [229]. Other NFAs, such as those based on IDT and IDTT fused-ring electron acceptors, also showed promise. Among these, the most notable is ITIC. This rigid molecule features a core structure of seven fused aromatic rings and typically crystallizes into structures exhibiting high electron mobility [229]. Consequently, when paired with a suitable polymer, ITIC-based OSCs can achieve high PCEs exceeding 11% [235]. The highest-performing polymer:NFA OSCs of this period were fabricated using a blend of the donor PBDB-T-SF and the acceptor IT-4F, reaching a PCE of 13.1% [236].

The next five years were predominantly marked by the emergence of a new NFA called Y6, which features an electron-deficient benzothiadiazole unit at its core. This unit promotes aggregation, and molecular packing, and even crystallization [229]. Indeed, Y6 and its derivative Y12 were recently shown to form single crystals of various shapes and sizes under specific SVA conditions, exhibiting intriguing absorbance properties [237]. Moreover, the central alkyl chains in Y6 are attached to the nitrogen atoms of the pyrrole units, which helps reduce voltage loss in Y6-based NFA OSCs, possibly by decreasing nonradiative recombination losses [229]. Consequently, different polymer:Y6 OSC combinations have achieved PCEs approaching 18.22% [99]. It is worth noting that other polymer:NFA blends, based on different donor polymers and NFAs, have been developed, but have not surpassed this PCE threshold. Nonetheless, some of these combinations have produced NFA-based OSCS with the highest recorded PCE of 10.24% for P3HT and a maximum PCE of 16.2% for a polythiophene derivative [99].

**Figure 9:**
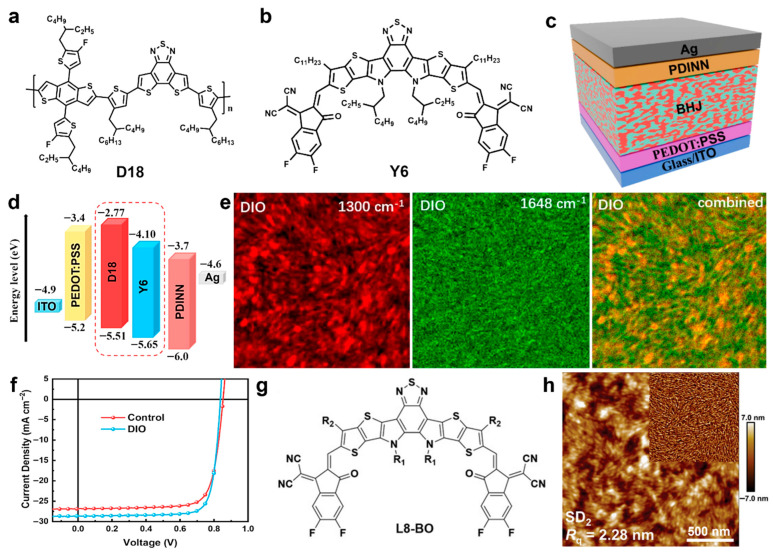
(**a**,**b**) Chemical structure of D18 (**a**) and Y6 (**b**). (**c**) Typical OSC device configuration. (**d**) Diagram depicting the energy levels of the D18:Y6 BHJ active layer. (**e**) A series of photo-induced force microscopy (PiFM) images emphasizing the microstructure of a D18:Y6 blend film processed with DIO additive. Here, a wave number of 1300 cm^−1^ represents Y6 and 1648 cm^−1^ represents D18, respectively. (**f**) J-V plots recorded for both control and DIO-processed OSC devices. (**g**) Chemical structure of L8-BO. (**h**) AFM height and phase (inset) images depicting the morphology of a D18:L8-BO film obtained after two-step processing via sequential deposition (SD_2_). Here, *R_q_* refers to the surface roughness. Reproduced from ref. [239] (**a**–**f**) [Copyright (2025) Elsevier B.V. All rights are reserved, including those for text and data mining, AI training, and similar technologies, with permission from Elsevier], and ref. [240] (**g**,**h**) [Copyright (2022) Wiley-VCH GmbH, with permission from John Wiley and Sons].

More recent examples of polymer:Y6 OSCs have demonstrated PCEs exceeding 19%. The first example is the PM6:Y6 blend. For example, Dong and coworkers processed the PM6:Y6 blend with a BDT additive, enhancing its aggregation and crystallinity properties. This improvement led to better exciton dissociation and charge transfer, more efficient carrier migration, and suppressed bimolecular recombination [84]. Consequently, the PM6:Y6 OSCs exhibited a PCE of 17.91%. When PM6 was replaced with D18 or its chlorinated derivative (D18-Cl), and BDT was substituted with DBTF and DBOF additives, the resulting OSCs achieved PCEs up to 19.19%. These high PCEs resulted from strong π–π coupling with Y6 in ordered, crystalline structures, which reduced non-radiative energy loss [238]. To the best of our knowledge, the record PCE in D18:Y6 (Figure 9a,b) binary OSCs (see a typical device configuration in Figure 9c) was very recently demonstrated by Zhao and coworkers [239]. They processed this blend, which exhibits suitable HOMO/LUMO alignments (Figure 9d), using a synergistic dual-phases morphology control protocol based on a novel solvent additive called DIO. This approach generated a nanophase-separated morphology composed of nanofibril networks that promote efficient charge transport (Figure 9e) [239]. When incorporated into OSCs, this morphology yielded a PCE of 19.35%. This PCE surpassed that of OSCs based on the control D18:Y6 bulk heterojunction (BHJ) processed without DIO (PCE = 17.76%; Figure 9f).

**Figure 10:**
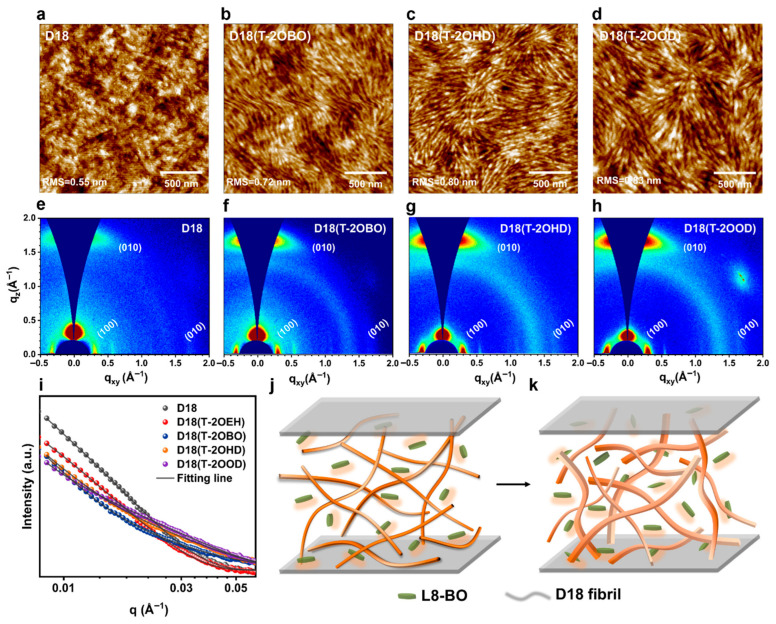
(**a**–**i**) AFM height images (**a**–**d**), 2D GIWAXS patterns (**e**–**h**), and 1D GISAXS profiles along the q*_xy_* axis (**i**) in D18 films before (**a**,**e**) and after processing (**b**–**d**,**f**–**h**) with T-2OBO (**b**,**f**), T-2OHD (**c**,**g**), and T-2OOD (**d**,**h**) additives. (**j**,**k**) Schematic diagram of the obtained morphology displaying thin fibrils in a D18:L8-BO film processed with T-2OEH (**j**), and thick fibrils in a D18:L8-BO film processed with T-2OOD (**k**). Reproduced from ref. [94] [Copyright (2024), with permission from the American Chemical Society].

Another NFA that has recently gained popularity in the fabrication of polymer:NFA OSCs is the so-called L8-BO. This NFA features a branched 2-butyloctyl alkyl chain and exhibits excellent absorption and packing properties. L8-BO (Figure 9g) has been shown to pair optimally with donor polymers such as D18 [240], MP6 [84], and PMQ-Si605 [241]. The resulting blends—D18:L8-BO, PM6:L8-BO, and PMQ-Si605:L8-BO—were demonstrated to form nanophase-separated ordered morphologies, after appropriate processing, including large nanofibers (Figure 9h) or fibrous structures. The corresponding OSCs exhibited PCEs of 19.05% [240], 19.01% [84], and 18.08% [241], respectively. Moreover, the D18:L8-BO blend was further optimized by modulating the active layer crystallization behavior using D18-Cl as a morphology modulator, which generated a well-structured nanofiber morphology that enhanced both charge separation and carrier transport [242]. The resulting dual-donor OSCs achieved a maximum PCE of 19.13%. This PCE was subsequently surpassed by PCEs recorded in OSCs based on D18:L8-BO blends that were (i) processed under high pressure [243], (ii) composed of both high and low molecular weight D18 [85], or (iii) processed with additives [94]. In the first case, processing the D18:L8-BO blend at elevated temperatures within a pressure-tight vial produced highly crystalline nanofibers with extended exciton diffusion lengths, resulting in a PCE of 19.65% in OSC devices [243]. In the second case, Wei and coworkers utilized both high and low molecular weight D18 donors to construct an ordered multiscale fibrous morphology, MIX-D18:L8-BO. This morphology was characterized by an enlarged exciton diffusion length, improved charge transfer and carrier migration, and reduced trap-assisted recombination, leading to OSCs with an excellent PCE of 20% [85]. In the third case, processing D18 with alkoxythiophene additives and TA yielded an ordered nanophase-separated morphology composed of nanofibrillar structures a few tens of nanometers wide and a few hundred nanometers long, exhibiting a dominant face-on orientation (Figure 10a–i) [94]. Consequently, the resulting D18:L8-BO fibrillar BHJ (Figure 10j,k)) not only promoted an efficient exciton dissociation but also facilitated charge transport, culminating in OSCs with an outstanding PCE of 20.1% [94].

Instead, the PM6:L8-BO blend has recently been successfully utilized at least twice in NFA-based OSCs to further improve the PCE from 19.01% [84] to 19.8% [94], and subsequently to 19.91% [244]. In the first example, PM6:L8-BO was processed with various alkoxythiophene additives and then subjected to TA to generate a nanophase-separated fibrillar morphology, resulting in OSCs with PCEs ranging from 19.3% to 19.8% [94]. In the second case, PM6:L8-BO-X blend was employed. L8-BO-X is a slightly modified version of L8-BO, characterized by a shift in the position of its branched side chains. This blend was processed using reversed TA to induce temperature-modulated nanophase aggregation, which produced honeycomb-like nanostructures. Consequently, while processing the PM6:L8-BO-X blend with conventional TA yielded OSCs with a PCE of 18.98%, using reversed TA increased the PCE of analogous OSCs to 19.91% [94].

Finally, we would like to mention that other efficient polymer:NFA blends have recently been processed using appropriate protocols to fabricate OSCs with PCE in the range of 19.2–19.53%. These include D18:Z19 [92], PBQx-TF:BTP-eC9-2Cl [245], and PM6:BTP-eC9 [246]. In the latter case, adding a dimer acceptor such as QD-1 to the PM6:BTP-eC9 blend can increase the PCE to 20.19%. This high PCE can be further improved to 20.6% by fabricating tandem OSCs [247].

### 5.3. Nanostructures Leading to High Power Conversion Efficiencies in All-Polymer OSCs

All-polymer OSCs (all-PSCs) have emerged as a promising alternative technology, offering excellent thermal stability, good processability, and relatively high PCEs. These advantages complement their already notable photostability, as well as exceptional device flexibility and stretchability. However, because all-PSCs are composed solely of polymeric materials, a significant drawback is their relatively low electron mobility, especially when compared to that of fullerenes or NFAs. Furthermore, while the molecular arrangements of polymer chains greatly influence the final efficiency of all-PSCs, controlling the microstructure in these systems remains challenging. In contrast, this issue is less pronounced in fullerene- and NFAs-based OSCs, where achieving good control over the blend microstructure is more feasible.

The first all-PSC was fabricated thirty years ago by Yu and Heeger using the donor MEH-PPV and the acceptor cyano--PPV (CN-PPV), achieving a modest PCE of 0.9% [248]. This low efficiency was most likely due to the relatively poor electron mobility of CN-PPV. Once this issue was addressed by designing new polymers (see Section 3 of this work), all-PSCs began to develop rapidly, especially after 2010. By 2018, the PCE of all-PSCs had improved significantly: from just over 1% when combining the donor PTB7 with the acceptor P(NDI2OD-T2) [249], to 5.7% when mixing PBDTTT-EF-T with P(NDI2OD-T2) [250], and exceeding 10% when blending PTzBI-Si with P(NDI2OD-T2) [251]. Subsequently, the morphology of the PTzBI-Si:P(NDI2OD-T2) blend was further optimized by manipulating solvent quality to enhance molecular co-facial packing, resulting in an increased PCE of 11% [252]. Comprehensive summaries of other polymer combinations and the progress of all-PSCs up to 2020 can be found in several detailed reviews available in the literature [134,146,230,253,254].

Between 2020 and 2022, all-PSCs achieved PCEs reaching the 15% limit. For example, Fan and coworkers utilized the donor polymerPBDB-T and a PF5-Y5 polymer to generate a fibrillar microstructure characterized by reduced aggregation and optimized phase separation. This morphology was expected to enhance exciton dissociation and charge transport. Indeed, the measured PCE of PBDB-T:PF5-Y5 all-PSCs was 14.45% [255]. A similar PCE of 14.4% was also achieved by blending PBDB-T with PJ1. The PBDB-T:PJ1 blend exhibited a microstructure consisting of a nanophase-separated interpenetrating network [256]. By optimizing the molecular weight of PBDB-T and subsequently applying TA, Zhang and coworkers improved the microstructure of the PBDB-T:PJ1 blend by enhancing its crystallinity and promoting a face-on orientation of polymer chains within the fibrillar microstructure [257]. This enhancement increased the PCE of PBDB-T:PJ1 solar cells to 15.4%. The PCE of all-PSCs was further improved to 15.8% through the incorporation of a novel narrow-band regioregular polymer acceptor based on benzotriazole (BTz)-core fused-ring segments, named PZT-γ [258]. The resulting morphology of the PBDB-T:PZT-γ active layer consisted of well-defined, phase-separated fibrous interpenetrating structures of optimal size, which promoted efficient exciton dissociation and effective charge transport [258].

Surpassing the 16% PCE threshold was achievable only in ternary all-PSCs utilizing the PM6 donor. This was accomplished either by combining PM6 with various electron acceptors, such as regioregular L15 and MBTI—synthesized through the copolymerization of dibrominated fused-ring electron acceptors with distannylated aromatic imide [259]—or by employing other donor:acceptors pairs, including PTQ10:PY-IT [260] and J71:PY-IT [261]. The highest PCEs obtained for these nanoscale phase-separated microstructures were 16.5% for the M6:L15:MBTI ternary blend [259], and 16.52% for the PM6:PTQ10:PY-IT [260] and PM6:J71:PY-IT blends [261].

Since 2023, various polymeric systems have been successfully employed to develop highly efficient donor-acceptor blends capable of achieving PCEs exceeding 19% in binary configurations [262]. Notably, all-PSCs with PCEs surpassing 17% were first reported for nanofibrillar morphologies, which promote binary exciton dissociation and enhance charge transport. These blends were created by mixing either the donor PBQx-TCl with the acceptor BTPIC*γ*-BDD [263], or the donor PBBTz-Cl with the acceptor PY-IT [264]. It is important to note that the donor PBQx-TCl results from the copolymerization of a chlorinated BDT-T unit with DTQx; PBBTz-Cl is a polymer synthesized by connecting the benzobisthiazole and chlorinated benzodithiophene units with thiophene, and BTPIC*γ*-BDD is based on the copolymerization of a BDD unit with an Y-shaped small NFA.

**Figure 11:**
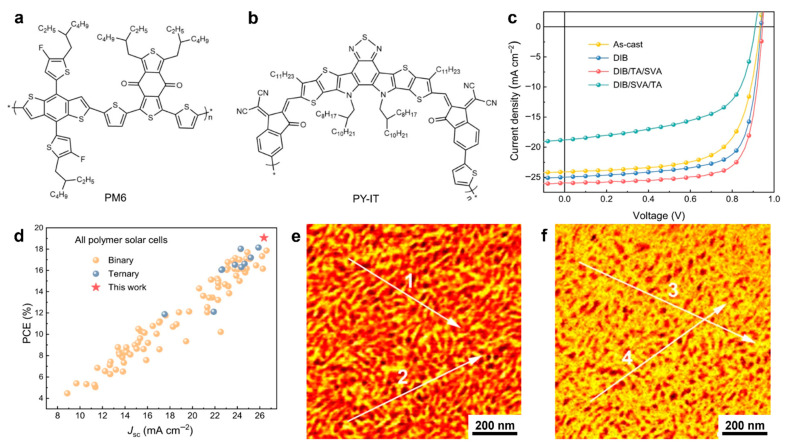
(**a**,**b**) Chemical structure of PM6 (**a**) and PY-IT (**b**). (**c**) *J-V* curves of all-PSCs processed in various conditions and measured under AM 1.5 G (100 mA/cm^2^). Here, DIB stands for the 1,4-diiodobenzene additive. (**d**) Comparison of the PCE versus *J*_SC_ for the most prominent all-PSCs reported in the literature until 2023. (**e**,**f**) Tapping AFM-based infrared spectroscopy micrographs recorded at the wavenumber of 1650 cm^−1^ (representing PM6) and 2215 cm^−1^ (representing PY-IT) for PM6:PY-IT blend films processed with DIB and exposed to TA and SVA. White arrows and numbers are not relevant here. Reproduced from ref. [262] [Copyright (2023) the author(s), with permission from Springer Nature].

Moreover, the PM6 donor (Figure 11a) appears to be the best match for PY-IT (Figure 11b), as demonstrated by the impressive device performance of all-PSC devices made from PM6:PY-IT blends (Figure 11c,d), with PCEs reaching up to 19.06% [262]. This record PCE was achieved by Zeng and coworkers, who (i) enhanced light collection by using a micro-sized surface topology and (ii) optimized the active layer by combining SVA with TA and the use of additives to control the kinetics of polymer crystallization. Through this processing, they generated a nano-to-micron sized hierarchical morphology composed of interconnected nanofibrillar donor-acceptor networks of molecular dimensions (Figure 11e,f) [262]. This morphology not only facilitated efficient photon-to-charge conversion, but also enabled a large light-receiving angle, resulting in a 30% increase in power output [262]. This record PCE of over 19% for binary (and ternary) all-PSCs (Figure 11d) was recently surpassed, approaching 20%, but only in all-PSCs based on multicomponent blends [83,265]. For example, Wang and coworkers diluted PM6 with PY-IT and subsequently diluted PY-IT with PM6 or D18 to generate highly structured donor-dominant or acceptor-dominant heterojunctions that enhanced charge carrier mobility and improved exciton diffusion length. These blends exhibited PCEs ranging from 18.8% to 19.4% [265]. Another example of multicomponent all-polymer blends was reported by Wu and coworkers, who employed a layer-by-layer (LbL) doctor-blading deposition technique to fabricate a quaternary PM6:D18-Cl:PY-SSe:PY-Cl structure. This architecture not only improved charge transfer and transport but also reduced losses caused by non-geminate recombination [83]. Consequently, all-PSCs based on LbL-processed PM6:D18-Cl:PY-SSe:PY-Cl structures achieved maximum PCEs of 19.46%, whereas their BHJ analogs exhibited lower PCEs of only 16.02% [83].

Finally, it is important to note that thermal stability remains a critical challenge for organic energy devices due to the inherently heat-sensitive nature of organic materials. Consequently, while polymer materials can degrade upon annealing at elevated temperatures, small semiconducting molecules may undergo volatilization and thermal oxidation. Moreover, both types of materials can experience morphological instabilities and changes in crystallinity, which may alter the microstructure and reduce the optoelectronic performance. For example, drawbacks of TA include electrode instabilities caused by the diffusion of metal atoms at high temperatures, unwanted interlayer reactions driven by thermal energy, and limited operational lifetimes, as organic energy devices exhibit performance degradation when subjected to prolonged TA at elevated temperatures. Potential solutions to these issues include the use of thermally stable materials (for example, polymers with high glass transition temperatures) and crosslinking polymer chains through chemical bonding.

**Table 3:** Summary of recent, most relevant photovoltaic properties reported in the literature for various types of OSCs. Most relevant values are marked in gray.

Donor:acceptor	Polymer Molecular Weight (kg/mol)	Nanostructure Type	Processing Method	J_SC_ (mA/cm^2^)	FF (%)	V_OC_ (V)	PCE (%)	Ref.
**Fullerene-based OSCs**								
P3HT:PC71BM	M_n_ = 7.15	single crystal	solution grown crystals/nanofibers were mixed with PC71BM and spin coated to fabricate thin films	7.69	54	0.59	2.45	[232]
P3HT:PC71BM	M_n_ = 7.15	nanofiber		5.93	45	0.57	1.52	[232]
P3HT:PC71BM	M_n_ = 48.8	single crystal		9.18	55	0.58	2.93	[232]
P3HT:PC71BM	M_n_ = 48.8	nanofiber		7.10	47	0.59	1.97	[232]
P3HT-*b*-PEG:PC71BM	M_n_ = 49.55	hairy single crystal		9.26	54	0.58	2.90	[232]
P3HT-*b*-PEG:PC71BM	M_n_ = 49.55	nanofiber		7.11	53	0.58	2.18	[232]
P3HT-*b*-PS:PC71BM	M_n_ = 7.669	hairy single crystal		7.74	51	0.58	2.29	[232]
P3HT-*b*-PMMA:PC71BM	M_n_ = 7.647	hairy nanofiber		5.47	47	0.58	1.49	[232]
PffBT4T-C_9_C_13_:PC71BM	-	38 nm polymer-rich domains comprising molecules adopting face-on orientations	spin coating from warm solutions	19.8	73	0.784	11.7	[93]
PM6:BTP-eC9:PC71BM multicomponent	-	~35 nm long nanofiber-like structures	spin coating solutions containing DQ	26.93	79.4	0.856	18.3	[234]
***OSCs based on non-fullerene acceptors***								
PBDB-T-SF:IT-4F	M_n_ = 20.9	nanophase-separated morphology	spin coating	20.88	71.3	0.88	13.1	[236]
PM6:Y6	-	morphology comprising homogeneous nanophase	processed with BDT additive, TA	27.61	77.11	0.841	17.91	[84]
D18:Y6 D18-Cl:Y6	-	small and homogeneous aggregation domains	processed with DBTF and DBOF additives, TA	27.65	77.99	0.890	19.19	[238]
D18:Y6	-	nanophase-separated morphology comprising nanofibril networks	synergistically dual-phases morphology control	28.6	80.84	0.836	19.35	[239]
D18:L8-BO	-	20 nm large nanofibers	spin coating sequential deposition	26.86	77.25	0.918	19.05	[240]
PM6:L8-BO	-	morphology comprising homogeneous nanophase	processed with BDT additive, TA	26.59	80.03	0.893	19.01	[84]
PMQ-Si605	M_n_ = 51.1	phase-separated fibrous structures	spin coating and TA	26.16	77.40	0.893	18.08	[241]
D18:L8-BO	M_n_ = 84.5	highly crystalline nanofibers	high-pressure fabrication in a pressure-tight vial	26.48	80.65	0.910	19.65	[243]
MIX-D18:L8-BO	M_n_ ≥ 40/<40	multiscale interpenetrating fiber network structure	spin coating and TA	26.75	81	0.920	20.0	[85]
D18:L8-BO	-	morphology comprising nanofibril aggregates with dominant face-on orientation	processed with alkoxythiophene additives, TA	26.5	81.2	0.906	20.1	[94]
PM6:L8-BO	-	phase-separated nanofibrillar morphology	processed with alkoxythiophene additives, TA	26.8	80.20	0.902	19.8	[94]
PM6:L8-BO-X	-	nanophase-aggregated honeycomb resembling nanostructures	reversed TA	28.12	79.46	0.891	19.91	[94]
D18:Z19	-	phase-separated fibrous morphology with preferential face-on orientation	spin coating and TA	24.6	77.60	1.002	19.2	[92]
PBQx-TF:BTP-eC9-2Cl	-	phase-separated fibrillar network with face-on orientation	processed with additives, TA	27.2	80.40	0.879	19.2	[245]
PM6:BTP-eC9 PM6:BTP-eC9:QD-1	M_n_ = 45	interpenetrating phase-separated fiber-like network structures	spin coating and TA	28.51 28.98	79.30 79.82	0.864 0.873	19.53 20.19	[246]
***All polymer OSCs***								
f-BTI2-FT:PTB7-Th	M_n_ = 13.8/-	phase-separated bicontinuous network	TA	11.55	57.04	1.04	6.85	[170]
PTzBI-Si:P(NDI2OD-T2)	M_n_ = 38.4/75.1	phase-separated crystalline morphology with preferential face-orientation	spin coating and TA	15.57	73.39	-	10.1	[251]
PBDB-T:PF5-Y5	M_w_ = -/33.3	fibral microstructures	spin coating and TA	20.65	74	0.946	14.45	[255]
PBDB-T:PJ1	M_n_ = -/23.3	finely nanoscale phase-separated interpenetrating network	spin coating and TA	22.3	70	0.9	14.4	[256]
PBDB-T:PJ1	M_n_ = 38/11.4	fibril-like nanostructures with preferential face-on orientation	spin coating and TA	22.7	75.3	0.9	15.4	[257]
PBDB-T:PZT-γ	M_n_ = -/7.8	phase-separated fibrous interpenetrating nanostructures	solvent optimization, spin coating and TA	24.7	71.3	0.896	15.8	[258]
PBBTz-Cl:PY-IT	M_n_ = 61.3/-	nanofibrillar D-A structures	spin coating and TA	24.56	73.73	0.947	17.15	[264]
PBQx-TCl:BTPIC*γ*-BDD	M_n_ = -/12.38	nanofibrillar D-A structures	spin coating and TA	24.1	76.92	0.944	17.5	[263]
PM6:PY-IT	-	interconnected nanofibrillar D-A networks	spin coating combined with SVA, TA, use of additives and topologically modified surfaces	26.37	76.48	0.945	19.06	[262]

## 6. Conclusions

In this review, we link the most relevant nanostructures fabricated in recent years from semiconducting polymers—using a variety of processing protocols—to their final optoelectronic properties (e.g., charge mobility, electrical conductivity, photovoltaic capabilities) measured in specific energy devices. We observe that ordered nanowires of PCDTPT, fabricated via liquid-bridge-mediated nano-transfer molding, exhibit the highest charge mobility among all semiconducting polymers (µ = 92.64 cm^2^V^−1^s^−1^, ref. [96]; note that ladder-type 2DCPs, excluded from this work, have demonstrated charge mobilities up to 970 cm^2^V^−1^s^−1^, ref. [169]). This dominance is shared by nanowires of DPPBTSPE, which show a hole mobility of 24 cm^2^V^−1^s^−1^ (ref. [29]), the highest value recorded for a DPP-based system. It is also noteworthy that nanofibers and fiber-like polycrystalline grains made of IID-based polymers can achieve efficient charge mobilities, with hole and electron mobilities reaching 14.4 cm^2^V^−1^s^−1^ and 9.7 cm^2^V^−1^s^−1^, respectively (refs. [95] and [149]). Furthermore, the highest electron mobility is that reported for F_4_BDOPV-2T structures (14.9 cm^2^V^−1^s^−1^, ref. [153]). In contrast, the highest mobilities for common polythiophene polymers remain those measured in highly ordered single crystals of P3OT (0.62 cm^2^V^−1^s^−1^, ref. [126]) and P3HT (0.5 cm^2^V^−1^s^−1^, ref. [90]). Finally, it is worth mentioning that charge mobilities can be significantly enhanced by chemical doping (for example, PEDOT doped with FeC_l3_ achieved mobilities exceeding 33 cm^2^V^−1^s^−1^, ref. [164]) and by blending with other polymers (for instance, nanofibers of PCDTPT mixed with PS experienced a hole mobility of 23.7 cm^2^V^−1^s^−1^, ref. [157]).

PEDOT fibrillar domains, displaying a face-on orientation and fabricated using the oCVD method combined with acid treatment, have achieved the highest electrical conductivity reported for a semiconducting polymer to date (6259 S/cm, ref. [182]). This conductivity significantly surpasses that of PANI self-assembled nanotubes (320 S/cm, ref. [220]) and that of ordered edge-on oriented crystalline structures of selenium-substituted DPP (977 S/cm, ref. [194]). Nanofibers of PEDOT:PSS, obtained by mixing PEDOT with the non-conductive PSS and processed via a solution shearing approach, exhibit a lower conductivity (4600 S/cm, ref. [187]) compared to the highest values recorded for PEDOT. However, this conductivity can be substantially increased to over 5000 S/cm by employing additives (ref. [188]). Furthermore, similar to charge mobility, electrical conductivity can be enhanced through chemical doping. For instance, doping a PBTTT derivative with FeCl_3_, combined with doctor blading, high-temperature film rubbing, and TA, produced π-stacked ordered domains of highly aligned polymer chains intercalated with dopants, resulting in an exceptional electrical conductivity of 2.2 × 10^5^ S/cm (ref. [205]).

The photovoltaic properties of semiconducting polymer-based nanostructures were summarized considering three main classes of OSCs. The first class, fullerene-based OSCs, has reached its performance limit in terms of PCE. For example, PffBT4T-C_9_C_13_:PC71BM OSCs fabricated from a BHJ comprising polymer-rich domains several tens of nanometers in size with molecules adopting face-on orientations, achieved a PCE of 11.7% (ref. [93]). Higher PCE values have only been reported for multicomponent OSCs, with the highest PCE of 18.3% observed in long PM6:BTP-eC9:PC71BM nanofiber-like structures containing DQ (ref. [234]). The second class, OSCs based on NFAs, has proven more versatile, with typical PCEs ranging from approximately 17% to just under 20%. Two recent studies demonstrated that D18:L8-BO multiscale interpenetrating fiber network structures and nanofibril aggregates with dominant face-on orientation, processed using alkoxythiophene additives, yielded OSCs with PCEs of 20% (ref. [85]) and 20.1% (ref. [94]), respectively. A higher PCE of 20.19% was recently achieved in OSCs based on interpenetrating phase-separated fiber-like network structures composed of PM6:BTP-eC9 blends containing additional dimer acceptors (resulting PCE = 20.19%, ref. [246]). It is also noteworthy that the *V*_OC_ of 1.002V measured in OSCs with D18:Z19 phase-separated fibrous morphology (ref. [92]) appears to be the highest reported to date. Finally, the third class comprises all-PSCs. Although reported PCEs for all-PSCs typically range between 15% and just under 18%, a breakthrough morphology consisting of PM6:PY-IT interconnected nanofibrillar D-A networks—fabricated by combining spin coating with SVA and TA, while employing additives and topologically modified surfaces—achieved a record PCE exceeding 19% (ref. [262]).

## Data Availability

Not applicable.

## Abbreviations

2DCP-MPc	2D phthalocyanine-based poly(benzimidazobenzophenanthroline);
2DCPs	two-dimensional conjugated polymers;
BDD	1,3-bis(thiophen-2-yl)-5,7-bis(2-ethylhexyl)benzo-[1,2-c:4,5-c′]dithiophene-4,8-dione;
BDT	benzo [1,2-b:4,5-b′]dithiophene;
BFDO	benzodifurandione;
BHJ	bulk heterojunction;
BPE	branched polyethylene;
BTBT	(di)thiophene, [1]benzothieno[3,2-b][1]benzothiophene;
BTP-eC9	2,2′- [[12,13-Bis(2-butyloctyl)-12,13-dihydro-3,9 dinonylbisthieno[2″,3″:4′,5′]thieno[2′,3′:4,5] pyrrolo[3,2-e:2′,3′-g][2,1,3]benzothiadiazole-2,10-diyl]bis[methylidyne(5,6-chloro-3-oxo-1H-indene-2,1(3H)-diylidene)]]bis [propanedinitrile];
BTP-eC9-2Cl	2,2′- [[12,13-Bis(2-butyloctyl)-12,13-dihydro-3,9-dinonylbisthieno[2″,3″:4′,5′] thieno[2′,3′:4,5]pyrrolo[3,2-e:2′,3′-g][2,1,3]benzothiadiazole-2,10-diyl]bis[methylidyne(2 or 3-chloro-3-oxo-1H-indene-2,1(3H)-diylidene) ]]bis [propanedinitrile];
C-AFM	conductive atomic force microscopy;
CDT	cyclopentadithiophene;
CDT-BTZ	polymer based on ecosyl-substituted cyclopentadithiophene-benzothiadiazole;
CN6-CP	hexacyano-trimethylene-cyclopropane;
CoCp_2_	bis(cyclopentadienyl)cobalt;
CPE	conjugated polyelectrolyte;
D-ODCB	deuterated 1,2 dichlorobenzene;
D-A	donor-acceptor;
D18	poly[(2,6-(4,8-bis(5-(2-ethylhexyl)-4-fluoro)thiophen-2-yl)-benzo[1,2-b:4,5-b′]dithiophene)-alt-5,5′-(5,8-bis(4-(2-butyloctyl)thiophen-2-yl)dithieno [3′,2′:3,4;2″,3″:5,6] benzo [1,2-c][1,2,5]thiadiazole)];
D18-Cl	poly[(2,6-(4,8-bis(5-(2-ethylhexyl)-4-chlorothiophen-2-yl)-benzo[1,2-b:4,5-b′]dithiophene))-alt-5,5′-(5,8-bis(4-(2-butyloctyl)thiophen-2-yl)dithieno[3′,2′:3,4;2″,3″:5,6]benzo[1,2-c][1,2,5]thiadiazole)];
DBOF	3,5-dibromotriffluoromethoxybenzene;
DBTF	3,5-dibromobenzotrifluoride;
DBTTT	dibenzothiopheno[6,5-b:6′,5′-f]thieno[3,2-b]thiophene;
DDB	dodecaborane;
DFSe	diketopyrrolopyrrole-difluorobenzoselenadiazole-diketopyrrolopyrrole;
DIO	1,8-diiodooctane;
DMSO	dimethyl sulfoxide;
DNTT	dinaphtho[2,3-b:2′,3′-f]thieno[3,2-b]thiophene;
DPP	diketopyrrolopyrrole;
DPP-2T	a polymer based on DPP and containing bithiophene;
DPPBTSPE	is based on DPP and 1,2-bis(5-(thiophen-2-yl)selenophen-2-yl)ethene (BTSPE);
DPPF-NTz	a polymer made of a furan-flanked diketopyrrolopyrrole (DPPF) as a monomer and napthobisthiadiazole (NTz) as comonomer units;
DTQx	dithieno[3,2-f:2′,3′-h]quinoxaline;
EMIM TFSI	1-ethyl-3-methylimidazolium bis(trifluoromethylsulfonyl) imide;
F4BDOPV-2T	a polymer composed of four-fluorinated benzodifurandione-based oligo(p-phenylene vinylene) (F4BDOPV) and 2,2′-bithiophene;
F_4_TCNQ	2,3,5,6-tetrafluoro-7,7,8,8-tetracyanoquinodimethane;
F8T2	poly(9,9-dioctylfuorene-co-bithiophene);
FBDPPV	a polymer system derived from fluorinated benzodifurandione (FBD);
FBDPPV-OEG	a polymer which incorporates oligo(ethylene glycol) (OEG)-substituted FBD;
Fe(OTf)_3_	iron(III) trifluoromethanesulfonate;
f-BSeI2TEG-FT	a polymer system obtained by selenium substitution in bithiophene imide;
f-BTI2-FT	a polymer based on a fused bithiophene imide (BTI);
f-BTI2TEG-T and f-BTI2TEG-FT	polymers comprising f-BTI2 bearing 3-(2-(2-methoxyethoxy)ethoxy)-2-((2-(2-methoxyethoxy)ethoxy)methyl)propan-1-yl side chain as the acceptor unit and thiophene/difluorothiophene as the donor co-unit (fluorine F atoms were introduced to further tune polymers′ frontier molecular orbital energy levels and crystallinity; *f*-fused);
GIWAXS	grazing incidence wide-angle X-ray scattering;
H_2_SO_4_	sulfuric acid;
HDPE	high-density polyethylene;
HOMO	highest occupied molecular orbital;
HOMPP	2-hydroxy-2-methyl propiophenone;
*I_D_*	drain current;
IDT	indacenodithiophene;
IDTT	indacenodithieno[3,2-*b*]thiophene;
IDTz	indacenodithiazole;
IID	isoindigo;
IT-4F	3,9-bis(2-methylene-((3-(1,1-dicyanomethylene)-6,7-difluoro)-indanone))-5,5,11,11-tetrakis(4-hexyl phenyl)-dithieno[2,3-d:2′,3′-d′]-s-indaceno[1,2-b:5,6-b′]dithiophene;
ITIC	3,9-bis(2-methylene-(3-(1,1-dicyanomethylene)-indanone))-5,5,11,11-tetrakis(4-hexylphenyl)-dithieno[2,3-d:2′,3′-d′]-s-indaceno[1,2-b:5,6-b′]dithiophene;
J71	poly[[5,6-difluoro-2-(2-hexyldecyl)-2*H*-benzotriazole-4,7-diyl]-2,5-thiophenediyl[4,8-bis[5-(tripropylsilyl)-2-thienyl]benzo[1,2-*b*:4,5-*b*′]dithiophene-2,6-diyl]-2,5-thiophenediyl];
L8-BO (also known as L8-BO-2F)	2,2′-((2Z,2′Z)-((12,13-bis(2-ethylhexyl)-3,9-(2-butyloctyl)-12,13-dihydro-[1,2,5] thiadiazolo[3,4-e]thieno[2″,3″:4′,5′]thieno[2′,3′:4,5]pyrrolo[3,2-g]thieno[2′,3′:4,5]thieno[3,2-b]indole-2,10-diyl)bis (methanylylidene))bis(5,6-difluoro-3-oxo-2,3-dihydro-1H-indene-2,1-diylidene))dimalononitrile;
LUMO	lowest unoccupied molecular orbital;
MEH-PPV	poly[2-methoxy-5-(2-ethylhexyloxy)-1,4-phenylenevinylene];
N-DMBI	4-(1,3-dimethyl-2,3-dihydro-1H-benzoimidazol-2-yl)-N,N-dimethylaniline;
n-PT4	a polymer system created by functionalizing each thiophene unit of a non-fused-ring polythiophene backbone with imide or cyano groups;
NFA	non-fullerene acceptor;
NDI	naphthalenediimide;
NH-FDPP-BT	fluorene-flanked diketopyrrolopyrrole (FDPP)-based semiconducting polymers containing bithiophene (BT) as the donor comonomers;
NH-FDPP-TT	fluorene-flanked diketopyrrolopyrrole (FDPP)-based semiconducting polymers containing thieno[3,2-b]thiophene (TT) as the donor comonomers;
NTz	napthobisthiadiazole;
oCVD	oxidative chemical vapor deposition;
OECTs	organic electrochemical transistors;
OFETs	organic field-effect transistors;
OLEDs	organic light-emitting diodes;
OSCs	organic solar cells;
P(g2T-T)	poly(2-(3,3′-bis(2-(2-(2-methoxyethoxy)ethoxy)ethoxy)-[2,2′-bithiophen]-5)yl thiophene);
P(g2T-TT)	poly(2--(3,3′--bis(2--(2--(2--methoxyethoxy)ethoxy)ethoxy)--[2,2′--bithiophen]--5--yl)thieno 3,2--b] thiophene);
P(gDPP-MeOT2), P(gDPP-T2) and P(gDPP-TT)	D-A copolymers based on 3,6-bis(5-bromothiophen-2-yl)-2,5-bis(2-(2-(2-methoxyethoxy)ethoxy) ethyl)-2,5dihydropyrrolo [3,4-c]pyrrole-1,4-dione;
P(gT2)	poly-[3,3′-bis(2-(2-(2-methoxyethoxy)ethoxy)ethoxy)-2,2′-bithiophene];
P(DPP-CNPz)	a polymer based on 3,6-dibromopyrazine-2-carbonitrile (CNPz);
P(DPP-DCNPz)	a polymer based on 3,6-dibromopyrazine-2,5-di-carbonitrile (DCNPz);
P(DPP-T)	poly(diketopyrrolopyrrole-co-thiophene);
P(NDI2OD-Se-Th 1.0)	poly[4-methyl-9-(5-(5-(9-(5-methylselenopheno[3,2-b]thiophen-2-yl)-2,7-bis(2-octyldodecyl)-1,3,6,8-tetraoxo-1,2,3,6,7,8-hexahydrobenzo[lmn][3,8]-phenanthrolin-4-yl)selenophen-2-yl)thiophen-2-yl)-2,7-bis(2-octyldodecyl)benzo[*lmn*][3,8]phenanthroline -1,3,6,8(*2H,7H*)-tetraone];
P(NDI2OD-T2) or N2200	poly{[N,N′-bis(2-octyldodecyl)-1,4,5,8-naphthalene diimide-2,6-diyl]-alt-5,5′-(2,2′-bithiophene)};
P(NDI2SiC6-T2)	a polymer composed of naphthalenediimide and bithiophene (T2) repeating units;
P(PzDPP-2FT)	a polymer featuring pyrido[3,4-b]pyrazine-diketopyrrolopyrrole (PzDPP) units and fluorinated bithiophene (2FT) blocks;
P(TDPP-BBT)	a D-A polymer based on thiophene-flanked diketopyrrolopyrrole and benzobisthiadiazole (BBT);
P(TDPP-BT)	a D-A polymer based on thiophene-flanked diketopyrrolopyrrole and benzothiadiazole (BT);
P(TDPP-TQ)	a D-A polymer (see its chemical structure in the inset) based on thiophene-flanked DPP and thymoquinone (TQ);
P(TzII-dTh-dTh)	a polymer synthesized by copolymerizing thiophene-flanked thiazoloisoindigo (TzII) with bithiophene;
P(TzII-dTh-dTz)	a polymer synthesized by copolymerizing thiophene-flanked TzII with bithiazole;
P2TDPP2TFT4	poly[((2,6--bis(thiophen--2--yl)--3,7--bis(9--octylnonadecyl)thieno[3,2--b]thieno[2′,3′:4,5] thieno[2,3--d]thiophene)--5,5′--diyl)(2,5--bis(8--octyl octadecyl)--3,6--di(thiophen--2--yl)pyrrolo[3,4--c]pyrrole--1,4--dione)--5,5′--diyl]];
P3HT	poly(3-hexylthiophene);
P3HT-*b*-PDL	poly(3-hexylthiophene)-*block*-poly(δ-decanolactone);
P3OT	poly(3-octylthiophene);
PA	polyacetylene;
PANI and TANI	polyaniline and tetraaniline;
PB	polybutadiene;
PBDB-T	poly[(2,6-(4,8-bis(5-(2-ethylhexyl)thiophen-2-yl)-benzo[1,2-b:4,5-b′]dithiophene))-alt-(5,5-(1′,3′-di-2-thienyl-5′,7′-bis(2-ethylhexyl)benzo[1′,2′-c:4′,5′-c′]dithiophene-4,8-dione))];
PBDB-T-SF	poly[(2,6-(4,8-bis(5-(2-ethylhexylthio)-4-fluorothiophen-2-yl)-benzo[1,2-*b*:4,5-*b*′]dithiophene))-*alt*-(5,5-(1′,3′-di-2-thienyl-5′,7′-bis(2-ethylhexyl)benzo[1′,2′-*c*:4′,5′-*c*′]dithiophene-4,8-dione)];
PBDP-F2	a polymer based on 3-dodecylthiophene-flanked (BDP) as the electron-acceptor moiety and thiophene derivatives containing fluorine atoms as electron-donor moieties;
PBDTTT-EF-T	poly[[4,8-bis[5-(2-ethylhexyl)-2-thienyl]benzo[1,2-b:4,5-b′]dithiophene-2,6-diyl][2-[[(2-ethylhexyl)oxy] carbonyl]-3-fluorothieno[3,4-b]thiophenediyl]];
PBFDO	poly(benzodifurandione);
PBQx-TF	a polymer that results from the copolymerization of a fluorinated benzo[1,2-b:4,5-b′]dithiophene (BDT-T) unit with dithieno[3,2-f:2′,3′-h]quinoxaline (DTQx);
PBTTT	poly(2,5-bis(3-alkylthiophen-2-yl)thieno[3,2-b]thiophene);
PCBM	phenyl-C61-butyric acid methyl ester;
PCE	power conversion efficiency;
PCDTPT	poly[4-(4,4-dihexadecyl-4H-cyclopenta [1,2-b:5,4-b′]dithiophen-2-yl)-*alt*-[1,2,5]thiadiazolo[3,4-c]pyridine];
PCDTPT-ODD	poly[4-(4,4-bis(2-octyldodecyl)-4H-cyclopenta[1,2-b:5,4-b′]dithiophen-2-yl)-alt-[1,2,5]-thiadiazolo [3,4-c]pyridine];
PCNI2-BTI	a polymer based on a cyano-functionalized fused bithiophene imide dimer;
PDFDSe	poly(4-(4,4-bis(2-ethylhexyl)-4*H*-silolo[3,2-*b*:4,5-*b*′]dithiophene-2-yl)-7-(4,4-bis(2-ethylhexyl)-6-(selenophene-2-yl)-4*H*-silolo[3,2-*b*:4,5-*b*′]dithiophene-2-yl)-5,6-difluorobenzo[*c*][1,2,5]thiadiazole;
PDMS	poly(dimethylsiloxane);
PDPP-DTT	polydiketopyrrolopyrrole-dithienylthieno[3,2-b]thiophene;
PDPP-TT-PDMS	poly-diketopyrrolopyrrole-thienothiophene-poly(dimethylsiloxane);
PDPP-TVT	poly[2,5-bis(2-decyltetradecyl)pyrrolo[3,4-c]pyrrole-1,4-(2H,5H)-dione-(E)-1,2-di(2,20-bithiophen-5-yl) ethene];
PDPP3T	poly(diketopyrrolopyrroleterthiophene);
PDPIN	a polymer based on the 2,2′-((2E,2′E)-(((2,5-bis(2-octyldodecyl)-3,6-dioxo-2,3,5,6-tetrahydropyrrolo[3,4-c] pyrrole-1,4-diyl)bis(thiophene-5,2-diyl))bis(methanylylidene))bis(3-oxo-2,3-dihydro-1H-indene-2,1-diylidene))dimalononitrile repeat unit;
PDPPT3	poly{2,2′-[(2,5-bis(2-hexyldecyl)-3,6-dioxo-2,3,5,6- tetrahydropyrrolo[3,4-c]pyrrole-1,4-diyl)dithiophene]- 5,5′-diyl-alt-thiophen-2,5-diyl};
PDPPT3_25%_	poly(2,5-bis (4-hexyldodecyl)-2,5-dihydro-3,6-di-2-thienyl-pyrrolo[3,4-c] pyrrole-1,4-dionealt-thiophene);
PDPPy-Se	a polymer based on pyridine and selenophene;
PDTTDPP	a copolymer composed of DPP and dithieno[3,2-b:20,30-d]thiophene (DTT);
PDTzSI-Se	a polymer which is based on thiazole imide with a high content of selenophene;
PEDOT	poly(3,4-ethylenedioxythiophene);
PE	polyethylene;
PEGDA	poly(ethylene glycol) diacrylate;
PF5-Y5	a polymer synthesized by coupling a (2,2′-((2*Z*,2′*Z*)-((12,13-bis(2-ethylhexyl)-3,9-diundecyl-12,13-dihydro[1,2,5]thiadiazolo[3,4e]thieno[2″,3″:4′,5′] thieno[2′,3′:4,5]pyrrolo[3,2-g] thieno[2′,3′:4,5]thieno[3,2-b]indole-2,10-diyl)bis(methanylylidene))bis(3-oxo-2,3-dihydro1H-indene-2,1-diylidene))dimalononitrile) (Y5) acceptor unit with a thienyl-benzodithiophene (BDT-T) donor unit;
PFClTVT	a polymer based on dichlorodithienylethene (ClTVT);
PffBT4T-C_9_C_13_	poly[(5,6-difluoro-2,1,3-benzothiadiazol-4,7-diyl)-alt-(3,3″′- di(2-nonyltridecyl)-2,2′;5′,2″;5″,2″′-quaterthiophen-5,5″′-diyl)];
PFO	poly(9,9-dioctylfluorene);
PgBTTT	a polymer obtained by relocating the glycol side chains position on the bithiophene unit of poly(2--(3,3′--bis(2--(2--(2--methoxyethoxy)ethoxy)ethoxy)--[2,2′--bithiophen]--5--yl)thieno 3,2--b] thiophene);
PJ1	poly[[12,13-bis(2-decyltetradecyl)-12,13-dihydro-3,9 diundecylbisthieno[2″,3″:4′,5′]thieno[2′,3′:4,5]pyrrolo[3,2-e:2′,3′-g][2,1,3]benzothiadiazole-2,10-diyl]methylidyne[1-(dicyanomethylene)-1,3-dihydro-3-oxo-2H-inden-yl-2-ylidene]-2,5-thiophenediyl[1-(dicyanomethylene)-1,3-dihydro-3-oxo-2H-inden-yl-2-ylidene]methylidyne];
PM6 (also known as PBDB-T-2F)	poly[(2,6-(4,8-bis(5-(2-ethylhexyl)-4-fluorothiophen-2-yl)-benzo[1,2-b:4,5-b′]dithiophene))-alt-(5,5-(1′,3′-di-2-thienyl-5′,7′-bis(2-ethylhexyl)benzo[1′,2′-c:4′,5′-c′]dithiophene-4,8-dione))];
PMMA	poly(methyl methacrylate);
PMQ-Si605	a polymer based on 6,7-difluoro-3-methylquinoxaline-thiophene backbone and containing 5% siloxane;
PNBDO-FDTE	poly((3E,7E)-3,7-bis(2-oxo-1Hpyrrolo[2,3-b]pyridin-3(2H)-ylidene)benzo[1,2-b:4,5-b′]-difuran-2,6 (3H,7H)-dione)-(E)-1,2-bis(3-fluorothiophen-2-yl)ethene;
PNDI-RO	a polymer consisting of *p*-conjugated backbones adjacent to alkyl chains;
PNDI2TEG-2Tz	a polymer system synthesized using bithiazole/2Tz donor units and NDI carrying triethylene glycol acceptors;
PNDIF-T2	poly[2,7-bis(11,11,12,12,13,13,14,14,15,15,16,16,17,17,18,18,18-heptadecafluoro octadecyl)-4-methyl-9-(5′-methyl-[2,2′-bithiophen]-5-yl)benzo[*lmn*][3,8]phenanthroline-1,3,6,8 (*2H,7H*)-tetraone];
PNDIF-TVT	poly[(E)-2,7-bis(11,11,12,12,13,13,14,14,15,15,16,16,17,17, 18,18,18-heptadecafluorooctadecyl)-4-methyl-9-(5-(2-(5-methylthiophen-2-yl)vinyl)thiophen-2-yl)benzo[*lmn*][3,8]phenanthroline-1,3,6,8(*2H,7H*)-tetraone];
PO12	a fused bithiophene imide dimer-based polymer incorporating distinct oligo(ethylene glycol) side chains;
PPE	poly(*para*-phenylene ethynylene);
PPV	poly(p-phenylene vinylene);
PPy	polypyrrole;
PQT	poly[5,50-bis(3-dodecyl-2-thienyl)-2,20-bithiophene)];
PS	polystyrene;
PSpF	poly(4-styrenesulfonyl fluoride);
PSS	polystyrene sulfonate;
PTB7	poly{[4,8-bis[(2-ethylhexyl)oxy]benzo[1,2-b:4,5-b′]dithiophene-2,6-diyl][3-fluoro-2-[(2-ethylhexyl)carbonyl] thieno[3,4-b]thiophenediyl]};
PTzBI-Si	poly{(4,8-bis(5-(2-ethylhexyl)thiophen-2-yl)benzo[1,2-b:4,5-b′]dithiophene-co-4,8-di(thien-2-yl)-2-(6-(1,1,1, 3,5,5,5-heptamethyltrisiloxan-3-yl)hexyl)-6-octyl[1,2,3]triazolo[4,5-f]isoindole-5,7(2H,6H)-dione};
PTB7-th	poly([2,6′-4,8-di(5-ethylhexylthienyl)benzo[1,2-b;3,3-b]dithiophene]{3-fluoro-2[(2-ethylhexyl)carbonyl] thieno[3,4-b]thiophenediyl});
PTIIG-Np	poly(thienoisoindigo-alt-naphthalene);
PTQ10	poly[[6,7-difluoro[(2-hexyldecyl)oxy]-5,8-quinoxalinediyl]-2,5-thiophenediyl]];
PTz-5-DPP	a polymer based on 3,6-di(thiazol-5-yl)-diketopyrrolopyrrole;
PU	polyurethane;
PU(DPP)_35_	a polyurethane multiblock copolymer containing a diketopyrrolopyrrole-based rigid block and a hydrogenated polybutadiene flexible block linked via urethane bonds;
PY-IT	poly[[12,13-bis(2-octyldodecyl)-12,13-dihydro-3,9-diundecylbisthieno[2″,3″:4′,5′]thieno[2′,3′:4,5] pyrrolo[3,2-e:2′,3′-g][2,1,3]benzothiadiazole-2,10-diyl]methylidyne[1-(dicyanomethylene)-1,3-dihydro-3-oxo-2H-inden-yl-2-ylidene]-2,5-thiophenediyl[1-(dicyanomethylene)-1,3-dihydro-3-oxo-2H-inden-yl-2-ylidene]methylidyne];
SBS	poly(styrene-*b*-butadiene-*b*-styrene);
SCLC	space charge-limited current;
SEBS	polystyrene-*block*-poly(ethyleneran-butylene)-*block*-polystyrene;
SNT	dithiophene-thiadiazolebenzotriazole;
SVA	solvent vapor annealing;
TA	thermal annealing;
T-2OBO	3,4-bis((2-butyloctyl)oxy)thiophene;
T-2OHD	3,4-bis((2-hexyldecyl)oxy)thiophene;
T-2OEH	3,4-bis((2-ethylhexyl)oxy)thiophene;
T-2OOD	3,4-bis((2-octyldodecyl)oxy)thiophene;
TAM	triaminomethane;
TDAE	tetrakis(dimethylamino)ethylene;
TDPP-Se	a copolymer composed of thiophene-flanked diketopyrrolopyrrole;
TFE	tetrafluoroethylene;
TfOH	trifluoromethanesulfonic acid;
ThDPP-CNBTz	a polymer which comprises a thiophene-flanked diketopyrrolopyrrole and a cyano functionalized benzothiadiazole;
TMB-PN	1,2,4-trimethylbenzene containing 1-phenylnaphthalene;
TNDP	thienyl naphthodipyrrolopyrrole;
TSA	p-toluene sulfonic acid;
TsOH	*para*-toluenesulfonic acid;
TTIF-BT	a donor-acceptor fused ring π-polymer featuring a dithioeno[3,2-b] thioenoindeno[1,2-b] fluorene (TTIF) backbone as the donor component;
TzII	thiophene-flanked thiazoloisoindigo;
*V_D_*	drain voltage;
*V_G_*	gate voltage;
XRD	X-ray diffraction;
Y12 (also known as BTP-4F-12 or Y6-BO)	2,2′-((2Z,2′Z)-((12,13-bis(2-butyloctyl)-3,9-diundecyl-12,13-dihydro-[1,2,5]thiadiazolo[3,4-e]thieno[2″,3″:4′,5′]thieno[2′,3′:4,5]pyrrolo[3,2-g]thieno[2′,3′:4,5]thieno[3,2-b]indole-2,10-diyl)bis (methanylylidene))bis(5,6-difluoro-3-oxo-2,3-dihydro-1H-indene-2,1-diylidene))dimalononitrile;
Y6 (also known as BTP-4F)	2,2′-((2Z,2′Z)-((12,13-bis(2-ethylhexyl)-3,9-diundecyl-12,13-dihydro-[1,2,5]thiadiazolo[3,4-e]thieno[2″,3″:4′,5′]thieno[2′,3′:4,5]pyrrolo[3,2-g]thieno[2′,3′:4,5]thieno[3,2-b]indole-2,10-diyl)bis(methanylylidene)) bis(5,6-difluoro-3-oxo-2,3-dihydro-1H-indene-2,1-diylidene))dimalononitrile;
Z19	a polymer based on a backbone containing alkoxy and phenyl-substituted alkyl chains.
